# Systematic Study of Quaternary Ammonium Cations for Bromine Sequestering Application in High Energy Density Electrolytes for Hydrogen Bromine Redox Flow Batteries

**DOI:** 10.3390/molecules26092721

**Published:** 2021-05-06

**Authors:** Michael Küttinger, Paulette A. Loichet Torres, Emeline Meyer, Peter Fischer, Jens Tübke

**Affiliations:** 1 Applied Electrochemistry, Fraunhofer Institute for Chemical Technology, Joseph-von-Fraunhofer Straße 7, D-76327 Pfinztal, Germany; michael.kuettinger@gmx.de (M.K.); paulette.loichet@tum.de (P.A.L.T.); la-emeline-54@hotmail.fr (E.M.); jens.tuebke@ict.fraunhofer.de (J.T.); 2 Institute for Mechanical Process Engineering and Mechanics, Karlsruhe Institute of Technology KIT, Straße am Forum 8, D-76131 Karlsruhe, Germany

**Keywords:** electrochemistry, bromine, quaternary ammonium salts, sequestration, hydrogen bromine redox flow battery, electrolyte, liquid/liquid phase equilibrium

## Abstract

Bromine complexing agents (BCAs) are used to reduce the vapor pressure of bromine in the aqueous electrolytes of bromine flow batteries. BCAs bind hazardous, volatile bromine by forming a second, heavy liquid fused salt. The properties of BCAs in a strongly acidic bromine electrolyte are largely unexplored. A total of 38 different quaternary ammonium halides are investigated ex situ regarding their properties and applicability in bromine electrolytes as BCAs. The focus is on the development of safe and performant HBr/Br_2_/H_2_O electrolytes with a theoretical capacity of 180 Ah L^−1^ for hydrogen bromine redox flow batteries (H_2_/Br_2_-RFB). Stable liquid fused salts, moderate bromine complexation, large conductivities and large redox potentials in the aqueous phase of the electrolytes are investigated in order to determine the most applicable BCA for this kind of electrolyte. A detailed study on the properties of BCA cations in these parameters is provided for the first time, as well as for electrolyte mixtures at different states of charge of the electrolyte. 1-ethylpyridin-1-ium bromide [C2Py]Br is selected from 38 BCAs based on its properties as a BCA that should be focused on for application in electrolytes for H_2_/Br_2_-RFB in the future.

## 1. Introduction

Within the last ten years, numerous chemical combinations of negative and positive half-cell chemistries for redox flow battery application have been published. The majority of these compositions have been investigated on laboratory scale [1,2,3]. One commercialized and noticeable redox flow battery system for stationary energy storage application is the hydrogen bromine redox flow battery (H_2_/Br_2_-RFB) [4,5,6].

One of the challenges attributed to the H_2_/Br_2_-RFB application is the high vapor pressure of bromine in the electrolyte solutions commonly used in this system [7]. In this study, 38 cost-effective additives based on quaternary ammonium halide salts are investigated for their applicability as bromine complexing additives (BCA) and their effects on electrolyte properties. The most promising additive is selected for further investigation in a cell. This systematic investigation is carried out for the first time for electrolytes in H_2_/Br_2_-RFBs.

### 1.1. State of the Art

In this flow battery system, the negative half cell resembles the negative half cell of a proton exchange membrane fuel cell anode, but is operated in a reversible mode. Liquid posolytes based on hydrobromic acid (HBr), bromine (Br_2_) and water (H_2_O) are pumped through the positive half cell, while bromide (Br^−^) is oxidized to Br_2_ during charge operation (Equation (1)) and vice versa during discharge operation [4,5,8]. In some research articles, gaseous positive half cells operating with bromine vapor are also discussed [4]. The thermodynamic cell voltage is 1.09 V [9]. Br_2_ itself is hardly soluble in water [10,11,12,13,14]. Nevertheless, if bromide ions are present, Br_2_ forms polybromides such as tribromide (Br_3_^−^), pentabromide (Br_5_^−^) or higher polybromides Br_2n+1_^−^ [13,15,16] following Equation (2):

2 Br^−^ ⇌ Br_2_ + 2 e^−^ (1)

Br^−^ (aq) + n Br_2_ (aq) ⇌ Br_2n+1_^−^ (aq) (2)

Bromine and bromide form addition bondings by complexation, resulting in the previously mentioned polybromide cations Br_2n+1_^−^ [17]. Complexation allows the maintaining of high concentrations of Br_2_ in aqueous bromide solutions. These electrolytes are particularly interesting for the application of Br_2_/Br^−^ electrolytes in RFBs due to their high theoretical energy density of up to 254 Wh L^−1^ and a theoretical volumetric capacity of up to 233 Ah L^−1^ (HBr 48 wt%). Ideally, in order to achieve high power densities in H_2_/Br_2_-RFBs, high concentrations of HBr and Br_2_ are indispensable [18]. Nevertheless, Br_2_ itself and in polybromide form is already quite volatile at ambient temperature [19,20,21] and can react with different electrode and cell components of the H_2_/Br_2_-RFBs [4,22,23,24,25,26]. One major concern is the crossover of Br_2_ from the positive to the negative half cell during cell operation. When Br_2_ comes into contact with the platinum-based catalysts in the hydrogen half cell, it can induce corrosive dissolution of platinum resulting in the formation of bromoplatinates [4,22,23,24,25,26]. The decay of the H_2_ electrode catalyst can be avoided by reducing the concentration of Br_2_ in the positive half cell electrolyte.

**Figure 1:**
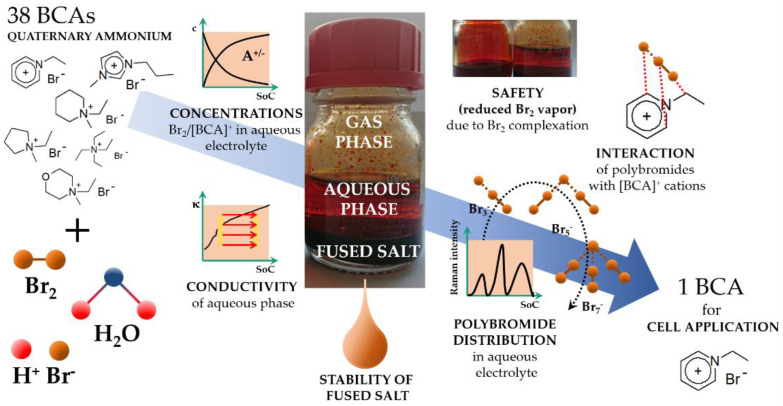
Scheme of the selection procedure of BCAs from 38 different compounds derived from six building blocks in an aqueous HBr/Br_2_ electrolyte. The selecting process includes the properties of primarily the aqueous electrolyte phase, such as safety, performance, stability, bromine sequestration and electrolytic conductivities. The results are used to draw conclusions about the interaction of the [BCA]^+^ cations with the polybromides Br_2n+1_^−^, which are used to explain the phenomena. On the basis of these results, one BCA out of 38 BCAs was selected for later investigations in the cell.

Stabilization of polybromides by conversion into a less volatile form would reduce the bromine vapor pressure, thus leading to a safer battery electrolyte [7]. Additives used to lower bromine’s volatility have been applied in zinc bromine RFB (Zn/Br_2_-RFB) [8,27,28,29,30,31,32,33,34,35,36,37,38,39] and vanadium bromine RFB (V/Br_2_-RFB) [40,41] electrolytes for a long time. These additives are mostly based on organic quaternary ammonium cation compounds and are called bromine complexing agents (BCA). Their bromide or chloride salts are usually highly soluble in aqueous electrolytes. However, their polybromide salts are only slightly soluble or even insoluble in aqueous media [33,36,42]. A further advantage of the polybromide salts of quaternary ammonium BCAs is their low melting point, leading to an insoluble ionic liquid at room temperature. In the electrolytes, these liquids form emulsions that coalesce over time by forming a heavy second phase in the electrolyte [36], as shown in Equation (3). For illustration, Figure 1 shows a photo of an electrolyte solution with a two-phase liquid Br^−^/Br_2_/H_2_O/[BCA]^+^ electrolyte in the center.

[BCA]^+^ (aq) + Br_2n+1_^−^ (aq) ⇌ [BCA]Br_2n+1_ (aq) ⇌ [BCA]Br_2n+1_ (fs) (3)

This heavy phase is often referred as the fused salt phase (fs), which sometimes tends to crystallize [31,42]. In the past, many BCAs have been evaluated for their application on zinc bromine batteries, out of which BCAs such as 1-ethyl-1-methylpyrrolidin-1-ium bromide [MEP]Br and 1-ethyl-1-methylmorpholin-1-ium bromide [MEM]Br [8,27,28,29,30,31,32,33,34,38,39,42,43] have received a lot of attention and have been investigated thoroughly. Other BCA structures such as heteroaromatic BCAs based on alkylated pyridine, picolines or imidazole [27,29,30,34,44], symmetrical and unsymmetrical alkylated aliphatic BCAs [33,40,42] and alkylated cyclic cations [27,29,36,42] have also been reported in the literature. Different side chains in N-position such as *n*-alkyl [27,29,31,33,34,36] or *iso*-alkyl groups [31] have been used to investigate their steric influence on melting point, complexation and solubility of the fused salt in Zn/Br_2_-electrolytes. 1-carboxymethyl [30], hydroxyalkyl groups [27,29] and 1-chloromethyl groups [33,36,42] have been identified to increase Br_2_ concentration in the aqueous phase in order to reduce precipitation of [BCA]Br_2n+1_. Polar side chain groups of BCAs have been found to also increase the solubility of the BCA in aqueous solutions. Mixtures of BCAs have been chosen to overlap the positive properties of different BCAs and overcome the drawbacks of single BCA properties [34,38,40,41,42,43].

Comprehensive work on BCAs has been carried out for Zn/Br_2_-RFB electrolyte mixtures and the properties have been described by Cathro et al. [42], Eustace [36] and Lancry et al. [31]. While the work on BCAs for Zn/Br_2_-RFB electrolytes is extensive and only a few studies applying BCAs in V/Br_2_-RFB have been published so far, no systematic study is available on the application of BCAs in HBr/Br_2_/H_2_O electrolytes for H_2_/Br_2_-RFB. In addition, the influence of the different polybromides on the electrolyte properties has not been investigated and is hardly documented in the literature for Zn/Br_2_-RFB [17].

### 1.2. Work Plan

In this work, we investigate 38 quaternary ammonium halides ex situ as prospective BCAs for their suitability for application in Br^−^/Br_2_ electrolytes. The aim is to obtain electrolytes for the positive half cell of a H_2_/Br_2_-RFB with a theoretical volumetric capacity of 180 Ah L^−1^. All investigated BCAs are shown in Table 1. They comprise of substances from six building blocks: (i) pyridines, (ii) pyrrolidines, (iii) morpholines, (iv) piperidines, (v) 3-methylimidazoles and (vi) tetraalkylammonium compounds. In this research article, the abbreviations listed in Table 1 are used for simplicity. In this study, the BCAs are examined for solubility in the electrolyte, the stability of a second liquid phase at room temperature and their bromine binding strength for a defined electrolyte composition. BCAs that do not form a stable liquid second phase are either not soluble in the electrolyte or show too low bromine binding strength and are excluded from a detailed study as they are not suitable for a battery application. BCAs, which are still of interest, are intensively investigated over the entire state of charge (SoC) range of the electrolyte with a focus on battery performance-relevant parameters: stability, concentration of Br_2_ in the aqueous electrolyte, redox potential as well as the electrolytic conductivity of the aqueous phase. For the first time, detailed investigations regarding parameter development are carried out on the basis of the measured distribution of Br_2_ on the polybromides tribromide (Br_3_^−^), pentabromide (Br_5_^−^) and heptabromide (Br_7_^−^) in the aqueous phase. Properties of the fused salt phase are not discussed in detail here in order to retain the clarity of the article. However, fused salt generally has low conductivity [36] and is therefore not the preferred option for cell applications. The criteria are defined to select a BCA for the application; the observed phenomena are explained in detail, and lastly, one BCA is selected for further use in a H_2_/Br_2_-RFB. The principle of this selection process is depicted in Figure 1.

## 2. Results and Discussion

### 2.1. Stabiltiy of Two-Phase Electrolytes and Bromine Binding Strength of BCAs

A total of 38 BCAs are synthesized for this work (marked with “(S)” in Table 1) or ordered from commercial suppliers (marked with “(C)” in Table 1). The component structure of the synthesized BCAs is detected and confirmed by means of ^1^H NMR and ^13^C NMR. NMR results of all synthesized components are available in the SM (Section 2) and are verified on the basis of NMR results from the literature [45,46,47,48,49,50,51].

#### 2.1.1. Solubility of [BCA]-Salts in Aqueous HBr Solutions

For their application on the H_2_/Br_2_-RFB, the bromide or chloride salts of the BCAs (Table 1) need to be soluble in aqueous acidic electrolyte at room temperature. This behavior is studied by a simple solubility test. For all investigated BCAs, a solubility test in electrolyte solutions with 1.11 M [BCA]X (X = Br^−^ or Cl^−^) and 5.47 M HBr in H_2_O is carried out at θ = 23 ± 1 °C. Most of the investigated substances are soluble in the electrolyte. Exceptions are the three BCAs [CTA]Br, [TOA]Br and [TOA]Cl. These compounds are not completely soluble in the mixture of HBr and H_2_O due to their long nonpolar alkyl side chains. Therefore, [CTA]Br, [TOA]Br and [TOA]Cl are not considered as BCAs for an application in a battery and are not investigated further in this work. All the other BCAs of Table 1 are soluble bromide or chloride salts and are investigated on their bromine binding strength and solubility properties related to the addition of Br_2_.

#### 2.1.2. Stability of Two-Phase Electrolytes and Bromine Binding Strength of BCAs

**Figure 2:**
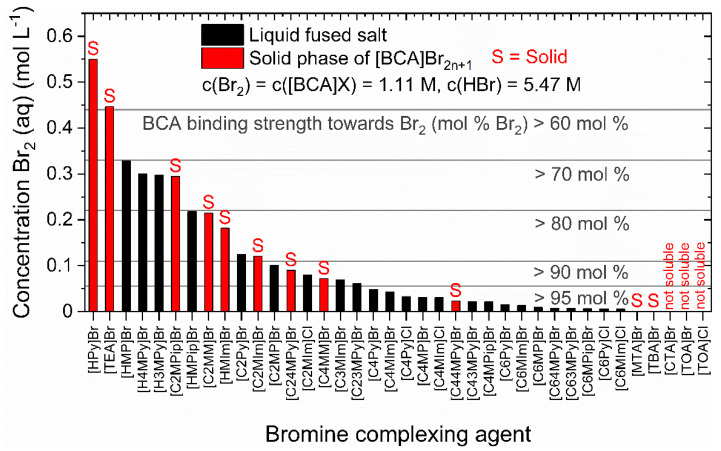
Concentrations of Br_2_ in the aqueous electrolyte phase and aggregate state of the fused salt (black bars = liquid, red bars = solid) of all BCAs shown in Table 1 for a selected state of charge (SoC) of 33% at θ = 23 ± 1 °C. The sorting is carried out for increasing bromine binding strengths (BCA complexation strength) of the fused salt. Non-soluble BCAs are shown on the right side. The binding strength (text in grey color) of the BCA indicates the fraction of Br_2_ related to 1.11 M Br_2_ bound in the fused salt at an SoC of 33%.

The soluble salts should form a liquid fused salt phase in a bromine electrolyte at room temperature, as crystallization of BCA salts in the pump or cell would lead to reduced volume rates and rising parasitic pressures. For the 35 soluble BCA salts, a complexation test in electrolyte solutions by adding 1.11 M Br_2_ was performed. The total electrolyte mixture corresponds to a mixture at an SoC of 33% (5.47 M HBr, 1.11 M Br_2_, 1.11. [BCA]X in H_2_O) at θ = 23 ± 1 °C. The SoC value is chosen, as it represents a 1:1 molar ratio of Br_2_ and BCA in the electrolyte. (A defintion of the SoC range is provided in the Materials and Methods section.) In the presence of aqueous polybromides, the BCA cations and the polybromides form either an orange-brown liquid fused salt phase or crystallize into reddish-brown or orange crystals. At the same time, a part of the Br_2_ remains in the aqueous electrolyte, resulting in a yellow to brown-colored aqueous electrolyte phase, depending on the applied BCA. The concentrations of Br_2_ in the aqueous solution and state of aggregation of the second bromine-rich, heavy phase of all BCAs are shown in Figure 2. The results in the figure are sorted according to the decreasing Br_2_ concentration in the aqueous electrolyte solution, which reflects the increasing strength of the BCA to bind Br_2_ in the second phase. The bromine binding strength of the BCA is defined by the molar fraction of Br_2_ stored in the fused salt phase compared to the total amount of Br_2_ in the sample. From left to right in Figure 2, the strength of the BCA in bromine binding increases. Additionally, the solubility of [BCA]^+^ cations in the aqueous phase in the presence of polybromides Br_2n+1_^−^ decreases from left to right following Equation (3). Fused salts that crystallize are shown as red bars, labelled by “S“, while stable liquid phases at room temperature are shown as black bars.

Figure 2 allows an initial classification of the BCAs with regard to the stability of their liquid fused salt phase and their binding strength towards Br_2_. The length of the alkyl side chain in N-position of BCAs has a decisive influence on the solubility of the cation in the presence of polybromide anions in the aqueous phase and on the stability of the fused salt. As the length of the alkyl side chain increases, bromine concentrations in the aqueous electrolyte phase (c(Br_2_(aq))) decrease, resulting in a rising binding strength of the BCA versus Br_2_/polybromides in the fused salt. Already for *n*-propyl, *n*-butyl and some BCAs with ethyl side chains in N-position, a concentration of c(Br_2_(aq)) < 0.1 M is present in the aqueous phase, whereby more than 91 mol% of Br_2_ is transferred into the fused salt. This increased binding of Br_2_ may increase the safety of the electrolyte, but it also sustains less Br_2_ in the aqueous electrolyte. This can lead to mass transport limitations during cell operation. Liquid fused salts with BCAs which are protonated in N-position [HMP]^+^, [H4MPy]^+^, [H3MPy]^+^ and [HMPip]^+^ show a lower bromine binding strength with Br_2_ concentrations between 0.22 to 0.33 M, which corresponds to a binding strength of only 70 to 80 mol% versus Br_2_ in the fused salt. For the right choice of BCA, a compromise between the safety due to strong Br_2_ binding property and the availability of polybromides in the aqueous electrolyte at the electrode has to be made. The aqueous phase is essential as it transports bromide, polybromides and protons, all required for the electrochemical reaction. The concentration values of Br_2_ in aqueous phases of the 35 samples are shown in detail in Figure S1 in the SM.

On the basis of our studies, we assume that the electron-donating inductive effect (+I-effect) influences the observed trend in Figure 2. The +I-effect increases with rising alkyl side chain length towards the nitrogen atom of the quaternary ammonium and leads to higher bromine binding strength with longer alkyl side chain BCAs and lower [BCA]Br_2n+1_ solubility in the aqueous electrolyte. In detail, due to the +I-effect, the polarity of the N atom is reduced, making the atom increasingly nonpolar and less soluble in the polar HBr/H_2_O environment as the chain length is increased. In these cases, stronger interactions with the polybromides might be given, while, due to reduced polarity, the interaction with Br_3_^−^, Br_5_^−^ and Br_7_^−^ is preferred compared to polar Br^−^ ions. To further explain the differences in bromine complexation with increased chain length, in particular on N-heterocycles, former studies on bromine interaction with quaternary ammonium ions can be considered. Easton et al. [17], for example, have calculated the interaction of Br_3_^−^ and Br_5_^−^ with different BCAs by DFT calculation and simultaneously determined the influence of polybromides on the hydrogen atoms in the region around the nitrogen atom in the BCA by ^1^H NMR spectroscopy. The protons in the α-positions of pyridinium, piperidinium and pyrrolidinium rings interact most intensively with the polybromide anion [17], which has also been shown by Lungwitz et al. for the application of polyiodines [52].

#### 2.1.3. Influence of Bromide or Chloride Counter Ion of the BCA on Bromine Binding Strength

Aqueous electrolytes containing BCAs with chloride counter ions have an increased bromine binding strength compared to the same [BCA]^+^ cations with bromide counter ions. They bind bromine 27.9 to 64.3% more strongly in the fused salt in comparison to the equivalent [BCA]^+^ cation with bromide anions. Next to [C2MIm]Cl, all further [BCA]Cl have a bromine binding strength of 93 mol% or higher, leading to a very low Br_2_ concentration in the aqueous electrolyte, which significantly limits their application in the RFB. Based on these results, this study concludes that chloride-based BCAs bind Br_2_ too strongly, yet a detailed study on the reason for this observation is not within the scope of this research article. It is only known in the literature that bromine forms with chloride mixed polyhalides such as ClBr_2_^−^ or ClBr_4_^−^ [53,54,55], which may have different properties when in contact with [BCA]^+^ cations. An evaluation of the differences between the complexation of polybromides or polyhalides for these electrolytes requires a further study.

### 2.2. Crystallization Behavior of Fused Salt Phases

#### 2.2.1. Influence of the Organic [BCA]^+^ Cation Structure on the Crystallization of Fused Salt

In order to understand the crystallization behavior of the various BCAs in the form of their fused salts, the structure of the BCA main component and the length of the alkyl side chain in N-position are considered. Crystallization of the fused salts of BCAs with N-hexyl side chains does not occur for all tested BCAs, and in the case of *n*-butyl substituents it occurs only sporadically. BCAs with an ethyl substituent ([C2MM]^+^) and 4-methylpyridin-1-ium compounds tend to crystallize more frequently due to less steric hindrance and a higher localized charge. Apart from [C2MIm]^+^, [C24MPy]^+^ and [C44MPy]^+^, the fused salt of all heteroaromatic BCAs alkylated in the N-position remains liquid at SoC = 33% and room temperature. The stability of their liquid phase at room temperature has also been shown in the literature for Zn/Br_2_-RFB electrolytes, but based on different electrolyte composition [27,29,30,34]. Reports on the precipitation of heteroaromatic BCAs could not be found previously in the literature. The previously mentioned [C2MIm]^+^, [C24MPy]^+^ and [C44MPy]^+^, however, offer a pronounced symmetry and predominantly short alkyl side chains. Protonated heteroaromatic BCAs ([HPy]Br, [HMIm]Br) without additional alkyl groups at the aromatic ring also tend to crystallize, while [H4MPy]Br and [H3MPy]Br form a liquid fused salt phase. A stable phase exists for [HMP]^+^ and [HMPip]^+^. Both of the latter remain liquid, and all stable BCAs protonated in the N-position have comparatively high Br_2_ concentrations between 0.2 M < c(Br_2_(aq)) < 0.35 M in the aqueous solution. Their bromine binding strength is between 70 and 80 mol% (Figure 2). All examined tetraalkylammonium bromides ([TEA]Br, [TBA]Br and [MTA]Br) are soluble in HBr/H_2_O but crystallize with polybromides under the given conditions. The symmetry of the BCA molecules leads to crystallization in H_2_/Br_2_-RFB electrolytes, which is also reported for this group in Zn/Br_2_ electrolytes with a different electrolyte composition [42].

In conclusion, organic BCA cations with a high symmetry in structure and short alkyl side chains tend to crystallize in contact with polybromides, while BCAs with long alkyl side chains and/or an unsymmetrical BCA structure stay liquid when forming a fused salt with polybromides.

#### 2.2.2. Polybromide Species in [BCA]Br_2n+1_ Crystals

**Figure 3:**
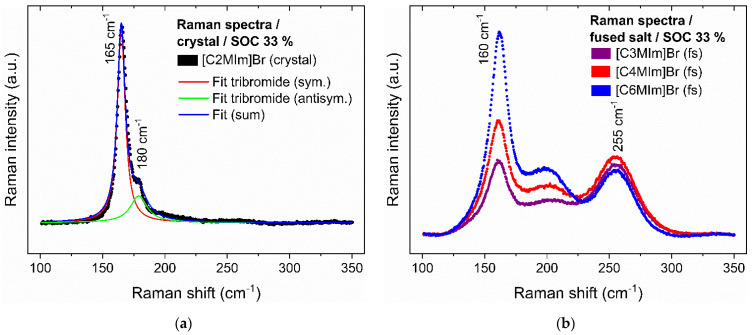
Raman spectra of (**a**) dry crystals of [C2MIm]Br_3_ including peak fitting for Raman shifts at ṽ = 165 cm^−1^ and ṽ = 180 cm^−1^ and (**b**) liquid fused salts of [C3MIm]Br_2n+1_, [C4MIm]Br_2n+1_ and [C6MIm] Br_2n+1_ at θ = 23 ± 1 °C each at an SoC of 33% (c(HBr) = 5.47 M, c(Br_2_)_total_ = c([BCA]Br = 1.11 M).

Beside the structure of BCAs, polybromides are found in the crystals. It raises the question of whether individual polybromides have a decisive influence on the tendency to crystallize with BCA cations. To investigate the reason behind the crystallization, Raman spectra of dry crystals are studied and are shown in Figure S2 of the SM for most of the [BCA]Br_2n+1_ crystals (except for [TBA]Br and [CTA]Br) and in Figure 3a using the example of the BCA [C2MIm]Br.

The Raman spectra of all show the same distinct features: a very strong peak in the Raman spectrum at a Raman shift (ṽ) between ṽ = 158–167 cm^−1^ and a weak peak at ṽ = 180–200 cm^−1^, which is an adjacent shoulder of the strong peak. An example of the Raman spectrum of [C2MIm]Br_3_ is shown in Figure 3a. Both peaks are characteristic for tribromide ions Br_3_^−^ due to its symmetrical (ṽ = 163 cm^−1^) and antisymmetrical (ṽ = 198 cm^−1^) stretching vibration known from [56,57,58]. The absence of water’s characteristic Raman bands, such as ṽ = 1600 cm^−1^ and between ṽ = 3000 cm^−1^ and 3800 cm^−1^ [59,60,61,62], ensures that dry crystals have been studied. Peaks of higher polybromides Raman shifts normally appear at ṽ = 210 cm^−1^ (Br_5_^−^, antisymmetric stretching vibration), ṽ = 253 cm^−1^ (Br_5_^−^, symmetric stretching vibration) and ṽ = 270 cm^−1^ (Br_7_^−^, symmetric stretching vibration) according to Chen et al. [56] but are not detected during the investigation of crystals. Higher polybromides, e.g., Br_5_^−^ or Br_7_^−^ are not present in the crystals.

However, higher polybromides seem to be bound in the liquid fused salts. The spectra of [C3MIm]Br_2n+1_, [C4MIm]Br_2n+1_ and [C6MIm]Br_2n+1_ are shown in Figure 3b, where the presence of pentabromide and probably heptabromide is evidenced by the appearance of a peak at ṽ = 255 cm^−1^. Figure 3b also shows an increase in peak intensity at ṽ = 160 cm^−1^ (Br_3_^−^) with increasing alkyl side chain length of the BCA, whereas the identified peak at ṽ = 255 cm^−1^ (Br_5_^−^/Br_7_^−^) remains constant. In general, the Raman peak shifts of the crystal samples are similar to those of the fused salts [58]. With an increasing side chain length at an SoC of 33%, bromine is preferentially added as Br_3_^−^ ions. This means that BCAs with longer side chains preferentially form a fused salt in which Br_3_^−^ acts as the counter ion to the [BCA]^+^ cation, but does not crystallize due to steric hindrance from the long alkyl side chain. On the other hand, fused salts formed from BCAs with short alkyl side chains are stable liquid phases when, due to a mixture of Br_3_^−^, Br_5_^−^ and Br_7_^−^, the fraction of higher polybromides increases in comparison to Br_3_^−^. In this case, the steric hindrance of the polybromides and charge distribution prevents the formation of crystals. The distribution of Br_2_ in the polybromides depends on the BCA and the specific interactions between BCA and the polybromides. Haller et al. [63] assume that higher polybromides are less strongly bound by BCAs than lower polybromides or bromide ions.

In summary, symmetric and small BCAs tend to crystallize at room temperature with Br_3_^−^ ions because of their higher charge density and lower steric hindrance. Both long side chains of heteroaromatic BCAs and large polybromides do not crystallize at room temperature due to steric hindrance. An exception is [C2Py]Br, which also remains liquid at room temperature.

For further consideration and application, therefore, N-alkylated pyridine-1-ium compounds and 1-alkylated 3-methylimidazol-1-ium compounds are of particular interest. With the alkyl side chains ethyl, *n*-propyl, *n*-butyl and *n*-hexyl, they all exhibit a bromine binding strength of more than 80 mol% at an SoC of 33% resulting in a reduced vapor pressure in the aqueous electrolyte solution. In order to compare their properties with well-investigated BCAs such as [C2MP]Br and [C2MM]Br, these two BCA groups are included in further experiments. In this first analysis, the focus has been set on the electrolyte mixture of 1:1 ratio of Br_2_ to BCA at an SoC of 33%. However, electrolytes at an SoC of 33% only provide a selective insight into the electrolyte system of a flow battery. In the following, the properties of the electrolytes are examined over the entire capacity range as a function of the SoC.

#### 2.2.3. Stability of Liquid Fused Salts within the Whole SoC Range

In the first preselection process, mainly heteroaromatic 1-alkylpyridin-1-ium and 1-alkyl-3-methylimidazole-1-ium bromides showed stable liquid fused salt phases at room temperature. This has been investigated for a fixed electrolyte mixture of 1.11 M [BCA]Br, 5.47 M HBr and 1.11 M Br_2_. To operate the battery with electrolytes containing BCAs, electrolyte stability over the entire SoC range between an SoC of 0% (7.6 M HBr, 1.11 M [BCA]Br and H_2_O) and an SoC of 100% (1 M HBr; 1.11 M [BCA]Br, 3.35 M Br_2_ and H_2_O) is required. Table 2 lists the investigated BCAs: [C2Py]Br, [C4Py]Br, [C6Py]Br, [C2MIm]Br, [C3MIm]Br, [C4MIm]Br and [C6MIm]Br, as well as [C2MM]Br (known as [MEM]Br) and [C2MP]Br (known as [MEP]Br), and indicates their usable SoC range for θ = 23 ± 1 °C.

**Figure 4:**
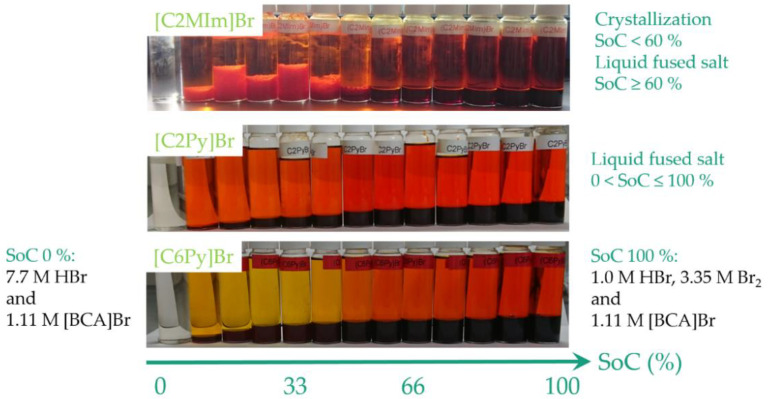
Photos of electrolyte samples with different BCAs [C2MIm]Br, [C2Py]Br and [C6Py]Br as a function of SoC for an SoC of 0, 10, 20, 30, 33, 40, 50, 60, 66, 70, 80, 90 and 100%. For electrolytes with [C2MIm]Br, between 0 < SoC < 60%, the second electrolyte phase is shown to precipitate and to form crystals. For [C2MIm]Br at SoC ≥ 60%, [C2Py]Br, as well as [C6Py]Br in the entire SoC range, the formation of a liquid fused salt phase occurs at room temperature. With increasing SoC, the volumes of the fused salt phase increase. In particular for the application of [C6Py]Br, the coloration of the aqueous phase from yellow to brown shows that the bromine concentrations increase with increasing SoC. In addition, the SoC range is specified by total concentrations for an SoC of 0% and 100%.

For different mixtures, it is shown that all BCAs form a second phase over the entire SoC range, except for an SoC of 0%. If Br_2_ is added to the aqueous solution, a separate fused salt phase is formed immediately. For all 1-alkylpyridin-1-ium compounds investigated for the entire SoC range, all fused salts remain liquid at room temperature over more than three years. With the exception of [C2MIm]Br, all investigated 3-methyl-imidazolium compounds form a liquid fused salt phase at room temperature within the entire SoC range. Electrolyte samples with the aqueous and fused salt phase in the whole SoC range for [C2MIm]Br, [C2Py]Br and [C6Py]Br are shown in Figure 4.

[C2MP]Br, [C2MM]Br and [C2MIm]Br form a solid crystalline phase with polybromides in the lower SoC range (Table 2). With these three BCAs, a maximum of 40 to 70% of the capacity range (71.8 to 125.7 Ah L^−1^) can be used at room temperature, as a crystallization of the fused salt cannot be accepted in the battery during operation. For [C2MM]Br, [C2MP]Br and [C2MIm]Br, dry crystals of the second phase in the lower SoC range are again examined by Raman spectroscopy. In the SoC range in which the fused salts tend to form crystals, only tribromide Br_3_^−^ salts of the BCAs are identified in addition to the characteristic peaks for the respective organic cation (Table 2).

In previous studies, [C2MM]Br and [C2MP]Br have been applied in electrolytes for Zn/Br_2_-RFB individually or in mixtures [8,28,30,33,38,41,42,43]. While the liquid fused salts of the Zn/Br_2_ electrolytes are stable at room temperature, these BCA form crystals for the selected acidic concentration at different SoC of a H_2_/Br_2_-RFB electrolyte. [C2MIm]Br, [C2MM]Br and [C2MP]Br are therefore excluded for use at room temperature and lower temperatures in H_2_/Br_2_-RFB since the formation of crystals in the cell or during operation can lead to strong overpressure in the system. [C2MM]Br and [C2MP]Br as BCAs are not investigated further in this study. On the other hand, [C2MIm]Br is reintroduced in the next experiments in order to compare its properties to the other derivatives such as [C3MIm]Br, [C4MIm]Br and [C6MIm]Br.

### 2.3. Bromine Binding Strength of [BCA]^+^(aq) Cations as a Function of the State of Charge (SoC)

**Figure 5:**
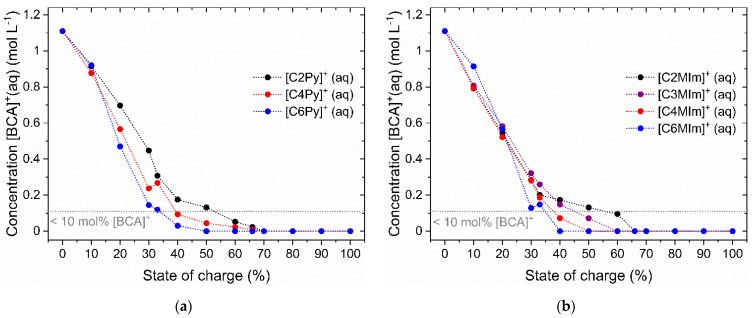
Concentration of [BCA]^+^ cations in the aqueous electrolyte phase containing (**a**) 1-alkylpyridine-1-ium bromides and (**b**) 1-alkyl-3-methylimidazole-1-ium bromides used as BCAs with different alkyl radicals in N-position as a function of the state of charge (SoC). All values of Figure 5 are printed in the SM in Table S1.

The bromine binding strength of the individual BCAs has been determined based on the concentration c(Br_2_) in the aqueous electrolyte solution compared to the total Br_2_ concentration at an SoC of 33% for the 35 chosen BCAs in Figure 5. Now, the change of the [BCA]^+^ concentration over the SoC range will be considered to investigate its effect towards the solubility and bromine binding properties for the seven BCAs based on 1-alkyl-1-pyridin-1-ium bromides and the 1-alkyl-3-methylimidazol-1-ium bromides. For the measurement of the BCA concentration in aqueous solution, the intensity of the characteristic peaks with corresponding Raman shifts is investigated. The Raman shifts of the [BCA]^+^ cations in aqueous solution are listed in Table 2. The Raman peaks selected for the evaluation have certain Raman shifts, which are highlighted in Table 2 in bold. The designated Raman shifts for the investigated BCAs are in agreement with the literature [64]. Concentrations of [BCA]^+^ cations in the aqueous phase are shown in Figure 5.

When the BCA is added to the polybromide solution the solubility equilibrium leads to a separation of [BCA]Br_2n+1_ salts by forming a fused salt phase according to Equation (3), or, as in the case of [C2MIm]Br, to the formation of crystals. The phase separation leads to a decrease in the concentration of [BCA]^+^(aq) regardless of the [BCA]^+^ cation type as shown in Figure 5. The concentration dependence of the [BCA]^+^ cations on the SoC shows a characteristic curve. At an SoC of 0% and thus c(Br_2_) = 0 M, a concentration of c([BCA]^+^) = 1.11 M is present, which decreases for all BCAs at SoC > 0%. Especially for 3-methylimidazol-1-ium derivatives, a linear and BCA-independent decrease in the [BCA]^+^ concentration between 0 ≤ SoC ≤ 20% can be observed (Figure 5b). For pyridin-1-ium derivatives, the same trend is observed between 0 ≤ SoC ≤ 10% in Figure 5a. Between 20 < SoC < 66%, the concentration of BCA continues to decrease for both groups of substances, although these changes become smaller as the SoC increases. In this range, the different bromine binding strength and solubility of the [BCA]^+^ cation with respect to the polybromide species become visible. [C6Py]Br and [C6MIm]Br show a considerably lower concentration in aqueous solution and therefore solubility as well as their property to bind bromine more strongly, respectively, than that of BCAs with shorter alkyl side chains. Organic cations of [C6Py]Br and [C6MIm]Br are no longer detectable in the solution even at SoCs of 50% and 40%, respectively. [C4Py]Br, [C4MIm]Br and [C3MIm]Br follow this trend at higher SoCs. [C2Py]Br and [C2MIm]Br show the lowest bromine binding strength versus polybromides. Thus, higher concentrations of [C2Py]^+^ and [C2MIm]^+^ remain in aqueous electrolytes at a higher SoC. From SoC ≥ 70% on, [C2Py]^+^ and [C2MIm]^+^ are not detectable in the Raman spectrum. Experimentally determined detection limits for [BCA]^+^ cations in aqueous solution are listed in Table 3/line 1. Due to an increasing total Br_2_ concentration with an increasing SoC, the [BCA]^+^ cations are gradually transferred into the fused salt phase. In principle, the SoC range can be divided into two sections: Depending on the [BCA]^+^ cation, there is a section for lower SoC values where [BCA]^+^ cations are present in the aqueous solution, and a section for higher SoC values, where [BCA]^+^ cations are not present in the aqueous solution but are completely present in the fused salt phase.

The individual, BCA-dependent bromine binding strength is determined from the [BCA]^+^ concentrations in the range of 20 ≤ SoC ≤ 60% and is applicable to investigate the solubility behavior of the BCA. The BCAs can be sorted according to their concentration in the aqueous phase. Low concentrations of [BCA]^+^(aq) in the aqueous phase describe a larger bromine binding strength and vice versa. The bromine binding strength is obtained in the following order: [C6MIm]^+^(aq) ≥ [C6Py]^+^(aq) > [C4MIm]^+^(aq) > [C4Py]^+^(aq) > [C3MIm]^+^(aq) > [C2MIm]^+^(aq) > [C2Py]^+^(aq). Correspondingly, the solubility of [BCA]Br_2n+1_ in the aqueous electrolyte for the 1-alkylpyridine-1-ium polybromides and for the 1-alkyl-3-methylimidazole-1-ium polybromides decreases with an increasing length of the alkyl side chain. The detection limits of [BCA]^+^ cations in Table 3 show that 1-alkyl-3-methylimidazol-1-ium is a slightly stronger bromine binding BCA than 1-alkylpyridin-1-ium if both have the same alkyl group in the N-position. As mentioned earlier, the bromine binding strength cannot be the only criteria to choose a BCA. Moderate Br_2_ concentrations in the aqueous phase along the whole SoC range are a prerequisite for the operability of the cell and will be investigated in the next section.

### 2.4. Effect of the Solubility Equilibrium on the Br_2_(aq) Concentration for the Whole SoC Range

**Figure 6:**
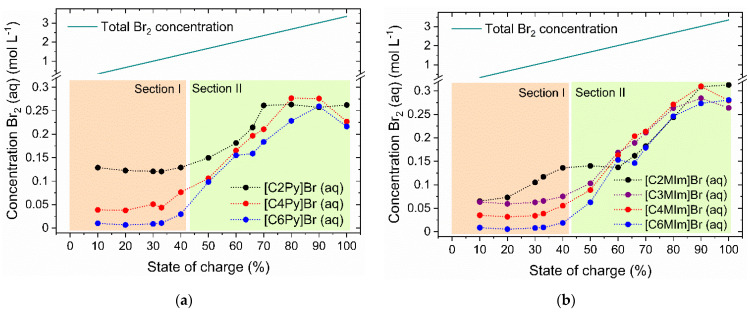
Concentrations of Br_2_ solved in the form of polybromide ions in the aqueous electrolyte phase depending on the state of charge (SoC) for the investigated BCAs: (**a**) 1-alkylpyridine-1-ium bromides and (**b**) 1-alkyl-3-methylimidazole-1-ium bromides as BCAs with different alkyl side chains in the N-position at θ = 23 ± 1 °C. All values of Figure 6 are printed in the SM in Table S2. Section I shows a strong dependence of the Br_2_ concentration in the aqueous phase on the alkyl side chain length of the BCA, while in section II the Br_2_ concentration is independent of the alkyl side chain length. The total Br_2_ concentration in the samples allows a direct comparison of the Br_2_ concentration in the sample and the aqueous phase for 10 ≤ SoC ≤ 100%.

Br_2_ is transported from the tank into the RFB half cell as an ingredient of the aqueous solution. Moderate Br_2_ concentrations dissolved as polybromide ions in the aqueous electrolyte phase are a prerequisite for stable cell operation. The bromine concentrations in the aqueous phase of the electrolytes for the investigated BCAs are plotted as a function of the SoC in Figure 6.

#### 2.4.1. General Influence of BCA Application on Br_2_ Concentration in Aqueous Solution

Due to the relatively low solubility of the BCA-polybromide salt, the Br_2_ concentration in the aqueous solution in equilibrium is low compared to the global concentration of Br_2_ indicated by the absolute concentration in Figure 6. While the total concentration of Br_2_ varies linearly along the SoC range, between c(Br_2_(total)) = 0.35 M at an SoC of 10% and c(Br_2_(total)) = 3.35 M at an SoC of 100% (Figure 6), the solubility equilibrium of the BCAs with the polybromides results in a significantly lower concentration of bromine (c(Br_2_(aq)) < 0.35 M) in the entire SoC range for all investigated BCAs. In addition, no linear relationship between c(Br_2_(aq)) and the SoC is observed. The maximum ratio of Br_2_ in the aqueous phase compared to the total Br_2_ concentration is present for SoC > 80% and is shown in Table 3/line 2. A maximum fraction of 11 mol% Br_2_ is present in the aqueous solution, while a fraction of more than 89 mol% Br_2_ is present in the fused salt phase. Although vapor pressures are not measured in this work, the safety of the electrolytes increases tremendously due to the low Br_2_ concentrations and the resulting reduced outgassing of Br_2_ from the aqueous electrolyte. Additionally, low concentrations of c(Br_2_(aq)) would decrease the available bromine that can crossover across the membrane to the H_2_ electrode inside the battery.

#### 2.4.2. Individual Concentration Trends of Different [BCA]^+^ Cations

For BCAs based on 1-alkylpyridin-1-ium and 1-alkyl-3-methylimidazol-ium, characteristic concentration curves for c(Br_2_(aq)) are obtained, whereby both diagrams in Figure 6 can be divided into two sections: In the first section (section I), between 0 < SoC ≤ 40% the Br_2_ concentrations in the aqueous solution are different for each BCA but are approximately independent of SoC and are also listed for an SoC of 30% in Table 3, on line 3. One exception is the trend of [C2MIm]Br, which will be explained later. There is a clear trend in Br_2_ concentrations in the aqueous phase at an SoC of 30% for the applied BCAs, which corresponds to the trend of BCA-concentrations in Section 2.3: c(Br_2_; [C6MIm]Br) < c(Br_2_; [C6Py]Br) < c(Br_2_; [C4MIm]Br) < c(Br_2_; [C4Py]Br) < c(Br_2_; [C3MIm]Br) << c(Br_2_; [C2MIm]Br) < c(Br_2_; [C2Py]Br). In this range, the bromine binding strength of the [BCA]^+^ cation is clearly visible again and corresponds exactly to the mentioned trends in Section 2.3: [C6MIm]^+^(aq) > [C6Py]^+^(aq) > [C4MIm]^+^(aq) > [C4Py]^+^(aq) > [C3MIm]^+^(aq) > [C2MIm]^+^(aq) > [C2Py]^+^(aq). The alkyl side chain length in the N-position in all BCAs is responsible for the differences in complexing strength, which is just a handy indicator for the solubility of the [BCA]Br_2n+1_. The longer the side chain, the lower the solubility of the [BCA]Br_2n+1_ in the aqueous solution. Another finding is that in general, the 1-alkyl-3-methylimidazol-1-ium compounds are slightly stronger BCAs compared to the 1-alkylpyridin-1-ium, when the same alkyl side chains are introduced. The concentrations of Br_2_ and [BCA]^+^ cations in the aqueous solution both show the same trend.

For the Br_2_ concentrations of [C2MIm]Br between 10 ≤ SoC ≤ 50%, there is a concentration plateau in Figure 6b which differs from the c(Br_2_(aq)) curves for [C3MIm]Br, [C4MIm]Br and [C6MIm]Br. As mentioned above, the second phase of [C2MIm]Br is crystalline and consists solely of the tribromide compound ([C2MIm]Br_3_). Consequently, the ratio of bromine to BCA cation is fixed to 1:1, and no higher polybromides can be stored in the second heavy phase. This leads to rising Br_2_ concentrations in the aqueous phase until the second phase liquefies again at SoC ≥ 50%, as presented in Figure 4.

The second section in Figure 6a,b starts from SoC > 40% (section II). Here the Br_2_ concentration increases noticeably for all BCAs, whereby the dependence of the Br_2_ concentration on the choice of the [BCA]^+^ cation decreases with increasing SoC. For both 1-alkylpyridin-1-ium and 1-alkyl-3-methylimidazolium cations as BCAs, the Br_2_ concentration for SoC ≥ 60% increases, whereby for each SoC value examined, approximately equal concentrations c(Br_2_(aq)) are present in the aqueous solution. In this section, the length of the alkyl side chain at the N-position of the BCA seems to have no influence on the binding strength of Br_2_ of the second phase in solution. However, 1-alkylpyridin-1-ium bromides bind bromine in section II slightly more strongly and tend to have a lower solubility in the presence of polybromides at SoC > 60% as slightly lower Br_2_ concentrations in the aqueous phase are present (Figure 6). In general, for SoC ≥ 80% the Br_2_ concentrations rise only slowly or form a plateau. In that range, the solubility of Br_2_ in the aqueous solution is limited by a lack of bromide ions to form polybromides, known from BCA-free electrolytes [65].

It is noticeable over the whole range that for small amounts of Br_2_ at SoC ≤ 40%, the complexation strength of the individual BCA types has a major influence on bromine concentration in the aqueous phase, but at SoC ≥ 60% neither the type of BCA base nor the length of the alkyl side chain seem to play a noticeable role, where all BCAs are considered to complex polybromides to a similar extent. At the same time, there is a transfer of Br_2_ from the aqueous phase into the fused salt over the entire SoC range, but since it has been shown above that no BCA cations are present in the aqueous solution at higher SoCs, the transfer no longer occurs according to Equation (3).

#### 2.4.3. Theoretical Comparison of Br_2_ Concentrations and Current Densities in the H_2_/Br_2_-RFB Cell

Overall, the concentrations of c(Br_2_(aq)) are concentrations of Br_2_ in chemical equilibrium for both phases and are only present during operation if the mass transfer of Br_2_ from the fused salt to the aqueous electrolyte phase takes place without any mass transfer limitation, i.e., the equilibrium state is maintained during the discharge process. Applying this theoretical assumption, the current densities of i = 723 mA cm^−2^ for c(Br_2_) = 0.3 M, i = 482 mA cm^−2^ for c(Br_2_) = 0.2 M and i = 241 mA cm^−2^ for c(Br_2_) = 0.1 M would be possible in a cell with a geometrically active electrode/membrane area of 40 cm² and a volume flow rate of 30 mL min^−1^. Data for this cell are described in [65]. For electrolytes with lower concentrations, the possible current densities would decrease further. Only [C2Py]Br forms a liquid fused salt over the entire SoC range and has concentrations c(Br_2_) ≥ 0.121 M in equilibrium between the two phases. In reality, lower concentrations can be expected in the aqueous solution. Therefore, a current density i < 250 mA cm^−2^ for galvanostatic charging and discharging can also be expected for electrolytes with [C2Py]Br. As [H4MPy]Br, [H3MPy]Br and [HMPip]Br upon first glance have aqueous Br_2_ concentrations between 0.20 M and 0.35 M at an SoC of 33% in Figure 2, they seem to be more applicable for effective cell operation. From Figure 6 it is noticed that in the range of an SoC of 33%, Br_2_ concentrations in the aqueous solutions are lowest. For the protonated forms it is therefore expected that for higher SoCs, much larger Br_2_ concentrations are present and electrolytes cannot still be classified as “safe” electrolytes. From the results in this section, it is expected that concentrations might rise for a higher SoC and lead to greater volatility of Br_2_. [C2Py]Br tends to be the [BCA] of choice in order to reach performance and safety of the Br_2_/Br^−^ electrolytes.

### 2.5. Distribution of Br_2_ on Polybromides in the Aqueous Phase

**Figure 7:**
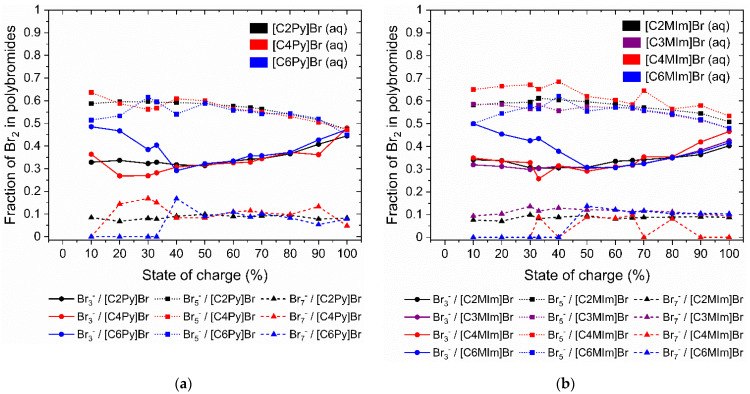
Distribution of Br_2_ in the aqueous electrolyte phase on the polybromides Br_3_^−^, Br_5_^−^ and Br_7_^−^, which are present, depending on the SoC with different BCAs with different alkyl side chains in the N-position for (**a**) 1-alkylpyridine-1-ium bromides and (**b**) 1-alkyl-3-methylimidazole-1-ium bromides as BCAs at θ = 23 ± 1 °C. All values of Br_2_ distribution on polybromides are printed in the SM in Tables S3–S5.

As presented in the previous section, the aqueous Br_2_ concentration defines the power capability of the cell. For a deeper understanding of the complexation behavior of the different BCAs, the Raman spectra of the aqueous phase have been analyzed regarding the distribution of bromine in the different polybromides. The method has been described in our earlier work [65]. The distribution of Br_2_ on Br_3_^−^, Br_5_^−^ and Br_7_^−^ is shown in Figure 7. The Raman spectra of all investigated samples are shown in the SM in Figures S3–S9.

An analysis of the distribution of Br_2_ among the polybromides Br_3_^−^, Br_5_^−^ and Br_7_^−^ in the aqueous phase shows basically similar results for both BCA groups as shown in Figure 7a,b. For all investigated BCAs, the storage of Br_2_ in the aqueous electrolyte in the form of Br_5_^−^ predominates, followed by storage in Br_3_^−^ and finally storage in Br_7_^−^. This is in accordance with earlier studies on pure BCA-free HBr/Br_2_/H_2_O-electrolytes [65], leading to the result that [BCA]^+^ has only a minor influence on the fractions of Br_2_ in the different polybromide species in the aqueous solution. For SoC < 40%, the *n*-hexyl side chain BCAs [C6Py]Br and [C6MIm]Br have a slightly increased Br_2_ content in Br_3_^−^, which means that less Br_2_ is distributed to the Br_5_^−^ and Br_7_^−^ anions. For SoC < 40%, *n*-butyl, *n*-propyl and ethyl BCAs have a constant Br_2_ distribution, which is independent of the BCA. For BCAs alkylated with an *n*-hexyl group, more Br_3_^−^ is available, while less Br_2_ is stored in Br_5_^−^ and Br_7_^−^. In this range, cations of [C6Py]^+^ and [C6MIm]^+^ are available in the aqueous solution and already prefer to interact with Br_3_^−^ in comparison to Br_5_^−^ and Br_7_^−^. This is in accordance with the composition of their fused salt phases, shown in Figure 3b.

For an SoC of ≥ 40%, there is no dependence of the Br_2_ distribution in the aqueous phase on the length of the alkyl radical present in the N-position of the BCA. The distribution is independent of the choice of the BCA basic compound pyridine or 3-methylimidazole. Within this SoC range, the fraction of Br_2_ stored as Br_3_^−^ increases from 30.7% to 41.6−47.3%, while proportionally less Br_2_ is stored in Br_5_^−^ and the curve decreases from 60% to 46–50%. Br_2_ remains in Br_7_^−^ within the whole SoC range relatively constant up to a maximum of 13.5%. Presumably, a better solvation of the more stable Br_3_^−^ compared to Br_5_^−^ is present. This effect is obtained also for BCA-free HBr/Br_2_/Br^−^ solutions [65]. Since the Br_2_ concentration of c(Br_2_(aq)) < 0.35 M and the distribution of Br_2_ to the polybromides is relatively constant in the whole range, no specific influence of this distribution on the performance or individual parameters is given and therefore cannot be discussed.

### 2.6. Influence of Different BCAs on Electrolyte Conductivity of the Aqueous Phase

**Figure 8:**
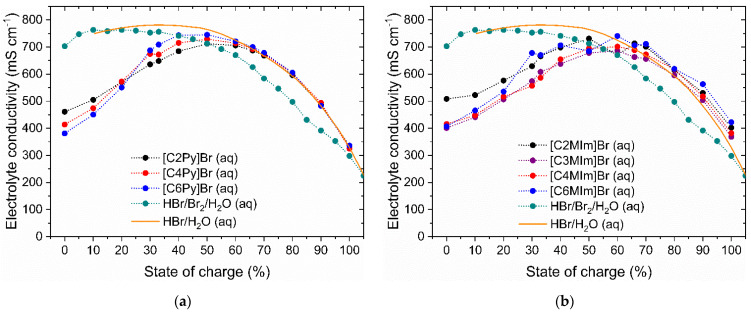
Specific electrolyte conductivity κ of the aqueous electrolyte phase containing (**a**) 1-alkylpyridin-1-ium bromides and (**b**) 1-alkyl-3-methylimidazol-1-ium bromides as BCAs with different alkyl side chains in the N-position as a function of state of charge SoC at θ = 23 ± 1 °C. In addition, electrolyte conductivities of BCA-free HBr/Br_2_/H_2_O electrolytes are shown (green dots) from [65] and of pure HBr/H_2_O solutions (orange line) from [66] at θ = 20 °C with the same total HBr and/or Br_2_ concentrations as reference curves.

For both flow-by and flow-through electrodes in RFBs, a low ohmic cell resistance is required to achieve high cell or stack performance. In RFB, this strongly depends on the electrolyte composition at different SoCs, usually targeting the maximum electrolyte conductivity of the aqueous electrolyte phase. Electrolyte conductivities of the aqueous phases from the seven pre-selected [BCA]/Br_2_ electrolytes summarized in Table 2 are shown in Figure 8. In addition, the reference conductivity curves of a BCA-free HBr/Br_2_/H_2_O electrolyte (from [65]) and a pure HBr/H_2_O acid solution within the SoC range are shown in Figure 8. All measured values are listed in Tables S6 and S7 in the SM. The values for pure HBr/H_2_O acid at θ = 20 °C are calculated from molar conductivities from [66] and are adapted to the SoC scale.

Electrolytic conductivities in the aqueous phase depend mainly on the high proton concentration and are larger than 300 mS cm^−1^ throughout the SoC range, whereby the Grotthus mechanism [67,68] is responsible for ionic transport through the solution. In this chain-hopping mechanism, bonds are attached and released between water and protons in oxonium ions. Ionic transport by the vehicle mechanism [67] is subordinated to the Grotthus mechanism in aqueous electrolyte solutions. With conductivities between 324.8 ≤ κ ≤ 745.0 mS cm^−1^, all aqueous electrolytes containing BCAs have large electrolyte conductivities and are feasible for application in the H_2_/Br_2_-RFB.

The [BCA]^+^ cations in the electrolyte mixture influence the electrolyte conductivity within the entire SoC range compared to BCA-free HBr/Br_2_/H_2_O electrolytes, as shown in Figure 8. For 0 ≤ SoC ≤ 60% for both 1-alkylpyridin-1-ium electrolytes and for 1-alkyl-3-methylimidazol-1-ium electrolytes, κ rises approximately from 368.90 to 478.00 mS cm^−1^ at an SoC of 0% to reach a peak at κ = 643.1−681.84 mS cm^−1^ between an SoC of 50 and 60%. Within this range, there is a difference from the two reference curves in accordance with the concentrations of the [BCA]^+^ cation in solution, shown in Figure 5. Initially, the organic character of [BCA]^+^ cation decreases the conductivity. Organic [BCA]^+^ cations with longer alkyl side chains lead to lower conductivity in the aqueous phase due to their increasing size. On the one hand, their migration in the electric field is slowed down and the ionic transport by the Grotthus mechanism is restricted. Grotthus charge transfer on a direct path between the electrodes is hindered and is forced to bypass the large organic cations. However, as the SoC is increased, the conductivity of the aqueous electrolyte in Figure 8 is shown to increase. This observation can be directly related to the results presented in Figure 5, whereby the [BCA]^+^ concentration of the investigated BCAs in the aqueous electrolyte is shown to decrease with the SoC as an indication that the cations are now bonded to the fused salt phase and leaving the aqueous electrolyte phase. Thus, based on these results, the decrease in the aqueous electrolyte [BCA]^+^ concentration translates into the increased electrolyte conductivity trend seen in Figure 8 between 0 ≤ SoC ≤ 60%.

For electrolytes with 1-alkylpyridin-1-ium bromide as a BCA, the complexation strength with respect to the side chain can be read in Figure 8a. Since [C6Py]^+^ with polybromides Br_2n+1_^−^ is less soluble in the aqueous phase than [C2Py]^+^, their concentration decreases more rapidly compared to [C2Py]^+^. The conductivity of the solution increases correspondingly more quickly in Figure 8a compared to electrolytes containing [C4Py]^+^ or [C2Py]^+^.

In the second range for 60 < SoC ≤ 100%, there are hardly any [BCA]^+^ cations available in the aqueous phase, as presented in Section 2.3. In this range, no direct dependence of conductivity on the alkyl side chain in the N-position of the [BCA]^+^ cation is obtained. Electrolytes with BCAs show approximately same conductivities. With an increasing SoC, the proton concentration decreases, and the conductivity of the solution decreases subsequently.

However, electrolytes containing BCAs reach higher conductivities compared to BCA-free HBr/Br_2_/H_2_O electrolytes for SoC ≥ 50% (green dots, in Figure 8). Due to the transfer of Br_2_ by use of the BCA from the aqueous phase into the fused salt, only concentrations lower than 0.35 M Br_2_ compared to the maximum total concentration of 2.99 M Br_2_ in BCA-free HBr/Br_2_/H_2_O electrolyte (calculated from values in [65]) are present in aqueous electrolytes. Since the absolute Br_2_ concentration in the aqueous electrolytes with BCA is much lower, while the concentration of protons in both solutions is assumed to be the same, the conductivity is higher when using BCAs and is comparable to pure HBr/H_2_O solutions as shown in Figure 8. The Grotthus mechanism takes place directly between the electrodes and does not need to bypass larger polybromides, as is the case in BCA-free HBr/Br_2_/H_2_O electrolytes [65]. As for SoCs > 60%, the [BCA]^+^ cations are not available in the aqueous phase; the conductivity of the resulting electrolytes in that range is shown to be higher than in BCA-free HBr/Br_2_/H_2_O electrolytes.

Overall, in the complete SoC range there are conductivities between a minimum of 324.8 mS cm^−1^ (SoC 100%) and a maximum of 712.0−745.0 mS cm^−1^ (an SoC of 50%, depending on the [BCA]^+^), which are large in comparison to aqueous electrolytes of Zn/Br_2_-RFB using the example of Eustace [36] with conductivities between 44.4 ≤ κ ≤ 128.2 mS cm^−1^ (θ = 23 °C). Pure aqueous solutions of comparable BCAs [C2MIm]Cl, [C4MIm]Cl and [C6MIm]Cl offer conductivities of κ ≤ 82 mS cm^−1^ at θ = 25 °C [69], whereby a clear dependence on the alkyl radical in the N-position of the 3-methylimidazole-1-ium can be confirmed from [69] with κ([C2MIm]Cl(aq)) > κ([C4MIm]Cl(aq)) > κ([C6MIm]Cl(aq)).

In this work, the achieved conductivities correspond to those of a pure HBr/H_2_O solution, which for an SoC of ≥ 60% means an increase in conductivity compared to HBr/Br_2_/H_2_O electrolytes and has a positive effect on cell performance. These high conductivities are due to the presence of the high proton concentration of c(HBr) ≥ 1 M. The influence of BCAs is directly attributable to the presence of BCAs for an SoC of < 60% and indirectly for an SoC of ≥ 60%. These high conductivities allow one to obtain high current densities at a low expected electrolyte overvoltage, depending on the half-cell structure.

### 2.7. Comparison of the Redox Potential of BCA-Free and BCA-Containing Electrolytes

To obtain a high-power density on an H_2_/Br_2_-RFB cell, a high thermodynamic cell voltage is beneficial. The thermodynamic cell voltage of the H_2_/Br_2_-RFB is composed of the difference of the half-cell redox potentials φ vs. NHE in open circuit state of the RFB. The redox potential of the positive half-cell φ(Br_2_/Br^−^) depends, in the simplest scenario, on the concentrations of the redox pair Br^−^/Br_2_ or Br^−^/Br_2n+1_^−^ according to Nernst [70], while assuming activity coefficients (γ) of γ = 1 for all components of the redox reaction according to Equation (4):

φ(Br_2_|Br^−^) = φ^0^(Br_2_|Br^−^) – 0.5 R T F^−1^ ln(c(Br^−^)^2^ c(Br_2_) ^−1^) (4)

**Figure 9:**
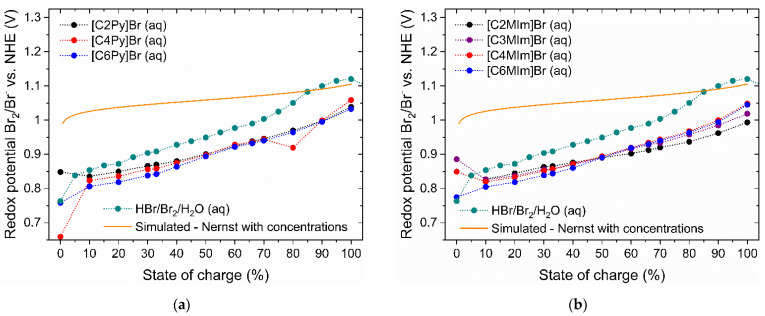
Redox potentials of the redox couple Br^−^/Br_2_ in aqueous posolyte vs. NHE as a function of its state of charge SoC measured on a glassy carbon electrode without [BCA]Br and with (**a**) 1-alkylpyridine-1-ium bromides and (**b**) 1-alkyl-3-methylimidazole-1-ium bromides as BCAs with different alkyl side chain groups in the N-position of the BCA at θ = 23 ± 1 °C. The orange line shows the simulated redox potential following the Nernst equation and is based on global concentrations of HBr and Br_2_. The measured redox potential of BCA-free HBr/Br_2_ electrolytes is shown with green dots for comparison and was first published in [70]. Values of redox potentials of the electrolytes containing BCAs are printed in the SM of this study in Table S8.

In Equation (4), F is the Faraday constant (96485 As mol^−1^), R the molar gas constant (8.3145 J mol^−1^ K^−1^) and T the temperature in K. φ^0^(Br_2_|Br^−^) is the standard redox potential of the Br_2_/Br^−^ electrolyte with 1.09 V [9]. The theoretical potential curve following the Nernst equation (Equation (4)) and global concentrations of HBr and Br_2_ at T = 298.15 K are applied. The curve is shown in Figure 9 as a function of the SoC (orange line). It has much higher values at corresponding SoCs compared to the measured redox potential of BCA-free electrolytes in Figure 9 (green dots). In the electrolyte system under investigation, there are no ideally diluted solutions, but strongly concentrated electrolytes. As a result, redox potentials according to the Nernst equation must be considered including activity coefficients as shown in Equation (5) [70]:

φ(Br_2_|Br^−^) = φ^0^(Br_2_|Br^−^) − 0.5 R.T.F^−1^ ln(c(Br^−^)^2^*γ*(Br^−^)^2^ c(Br_2_)^−1^*γ*(Br_2_)^−1^) (5)

For low SoCs with high concentrations c(HBr) > 1.2 M of HBr, the activity coefficients for γ(HBr) > 1 are available (calculated with data from [71]). Increasingly higher activities a(HBr) = c(HBr) γ(HBr) must be taken into account in the calculation of the redox potential of the positive half cell due to constantly increasing activity coefficients of HBr in the aqueous solution [71]. Wlodarczyk et al. [70] clearly show that the increasing activity coefficients of HBr for low SoCs leads to a strong decrease in the redox potential of the bromine electrolyte at an electrode according to Nernst compared to a pure concentration-based calculation.

The measured redox potential φ(Br_2_/Br^−^) of the pure HBr/Br_2_/H_2_O electrolytes (green curve in Figure 9) increases with an increasing SoC from 0.76 V vs. NHE (at an SoC of 0%) and a maximum value at an SoC of 100% at 1.12 V vs. NHE. For SoC ≥ 89.4%, Br_2_ is not completely soluble in the aqueous solution as explained in [65]. The aqueous solutions contain less Br_2_, and the redox potential flattens following the Nernst equation (Equation (5)).

In principle, the redox potential of the aqueous electrolyte solution with the use of [BCA]Br in the presence of Br_2_ is lower within the entire SoC range compared to BCA-free HBr/Br_2_/H_2_O electrolytes (Figure 9). The transfer of Br_2_ into the fused salt reduces the bromine concentration in the aqueous solution, while at the same time the HBr concentrations stay nearly constant in comparison to BCA-free HBr/Br_2_/H_2_O electrolytes. Hence, according to Equation (4), the redox potential of [BCA]Br electrolytes is slightly lower when compared to the BCA-free HBr/Br_2_/H_2_O electrolytes. For SoCs < 50%, the solubility of the respective [BCA]^+^ cation is responsible for the trend observed in Figure 9. In accordance with lower bromine concentrations in the electrolyte for increasing the *n*-alkyl side chain length (Figure 6), lower redox potentials are observed with the BCA electrolytes with larger *n*-alkyl chain. For SoCs > 50%, the difference between the potentials of the pure HBr/Br_2_/H_2_O electrolyte and the electrolytes containing BCA becomes increasingly larger. In global terms, the Br_2_ concentration increases strongly, which can be seen in the potential of the BCA-free HBr/Br_2_/H_2_O electrolyte, but at the same time the majority of the Br_2_ in the solutions with BCAs is transferred to the fused salt. The redox potentials of the electrolytes with BCAs increase at a slower rate with an increasing SoC.

The use of [BCA]Br leads to an average reduction in the redox potential of the positive half cell and the cell voltage ΔE by 32 ≤ ΔE ≤ 114 mV for all BCAs and over the entire SoC range compared to pure HBr/Br_2_/H_2_O electrolytes. The performance of the cell is reduced by this effect, while it is already lowered by high activity coefficients of HBr [70,71]. Fabjan et al. [8] described for Zn/Br_2_-RFB electrolytes that solutions of [C2MM]Br and [C2MP]Br reduce the concentration of Br_2_ in the aqueous phase to approximately 0.01 M, and the redox potential is characteristically reduced by 50 to 70 mV. This applies to our electrolytes and both BCA series in Figure 9 for the range 30 ≤ SoC ≤ 70%. A lower cell voltage due to the use of BCAs must be accepted as a trade-off for limiting the Br_2_ concentration in the aqueous solution.

### 2.8. Interpretation of the Study and Selection of a Promising BCA for Application in Cell Tests

In the scope of this research, 38 cost-effective BCAs have been investigated and evaluated on the basis of their solubility, Br_2_ binding strength, ionic conductivity and equilibrium potential for their possible application as electrolytes on H_2_/Br_2_-RFBs. In the following, an elimination procedure is applied to select the most suitable BCA for battery operation. The BCAs are excluded on the basis of the results obtained in the previous sections. To start, based on their solubility, [CTA]Br, [TOA]Cl and [TOA]Br do not dissolve in a polar HBr/H_2_O solution due to their long nonpolar alkyl groups. In addition, other BCAs such as [HPy]Br, [TEA]Br, [C2MPip]Br, [C2MP]Br, [C2MM]Br, [C4MM]Br, [HMIm]Br, [C2MIm]Br, [MTA]Br, [TBA]Br, [C24MPy]Br and [C44MPy]Br form solid crystals in the aqueous solution instead of a liquid fused salt when they come into contact with polybromides. Hence, none of these BCAs can be used in the cell as the spontaneous formation of crystals in the cell, tubing and pumps can lead to operational problems.

Since the H_2_/Br_2_ RFB is intended to be operated at high current densities due to the good reaction kinetics of both electrode reactions, a high mass flow of Br_2_ into the cell during the discharge reaction is necessary, making high concentrations of Br_2_ desirable. At the same time, it is the task of the BCAs to reduce the bromine concentration in the aqueous phase in order to increase safety. All BCAs that form a liquid fused salt phase and have long alkyl side chains such as *n*-butyl or *n*-hexyl have a bromine binding strength of >95 mol%. Additionally, all BCAs with a liquid fused salt phase and ethyl or *n*-propyl as a side chain, with the exception of [C2Py]Br, have a bromine binding strength of >90 mol%. These BCAs do not leave sufficient Br_2_ in the aqueous phase of the electrolyte to achieve acceptable current densities. [HMP]Br, [H4MPy]Br, [H3MPy]Br and [HMPip]Br show only a bromine binding strength of 70 to 80 mol% at an SoC of 33% with concentrations between 0.22 to 0.33 M, while it is expected that for higher SoC values, larger Br_2_ concentrations than 0.5 M are available. The essential safety might not be achieved at these concentrations.

It is therefore proposed to investigate 1-ethylpyidin-1-ium bromide [C2Py]Br as BCA in further studies in the RFB cell. From the seven BCAs investigated over the entire SoC range, [C2Py]Br exhibits the most advantages. Even though there is a reduction in the electrolyte conductivity and the redox potential when compared to the BCA-free one, the concentrations of Br_2_ in the aqueous solution are higher compared to solutions with [C4Py]Br and [C6Py]Br. In the [C2Py]Br, electrolytes at least 89 mol% Br_2_ remain in the fused salt phase, yet the electrolytes still maintain large Br_2_ concentrations, between 0.12 M to 0.26 M, in the aqueous solution. The availability of Br_2_ in the liquid electrolyte phase turned out to be a decisive factor in our selection, making this compound clearly stand out over all the other investigated BCAs. The initial cycling tests and investigation of the [C2Py]^+^ cations on the cell performance during the charge and discharge operation are shown in parallel in [72].

## 3. Materials and Methods

A detailed description of the testing methods is given in the SM (Section 1) for this article to enable reproducibility of the measurements. An abbreviated form is reproduced here.

### 3.1. Reagents

Reagents used for preparation of bromine electrolyte samples in this work are hydrobromic acid, bromine and distilled water. Chemicals for the synthesis of BCAs are named in the Supplementary Materials (SM) including suppliers and purities.

### 3.2. Synthesis of Bromine Complexing Agents

The investigated BCAs are listed in Table 1, including chemical structure, name and abbreviation. Non-commercially available N-alkylated quaternary ammonium bromide salts based on pyridine ([C2Py]Br, [C4Py]Br, [C6Py]Br, [C6Py]Cl), on 3-picoline ([C23MPy]Br, [C43MPy]Br, [C63MPy]Br), on 4-picoline ([C24MPy]Br, [C44MPy]Br, [C64MPy]Br), on 1-methylimidazole ([C2MIm]Br, [C3MIm]Br, [C4MIm]Br, [C6MIm]Br, [C6MIm]Cl), on 1-methylpiperidine ([C2MPip]Br, [C4MPip]Br, [C6MPip]Br,) and on 1-methylpyrrolidine ([C6MP]Br) are synthesized by alkylation reaction [45,73] according to Dzyuba et al. [74]. Some BCAs are available as golden liquids at reaction temperatures between 40 °C and 100 °C, while some precipitate as golden or white solids. ^1^H NMR and ^13^C NMR spectra of the synthesized substances are presented in SM and are consistent with NMR shifts found in the literature [45,46,47,48,49]. Protonated forms of 4-picoline, 3-picoline, 1-methylpiperidine, pyridine, 1-methylimidazole and 1-methylpyrrolidine ([H4MPy]Br, [H3MPy]Br, [HMP]Br, [HMPip]Br, [HPy]Br, [HMIm]Br) are prepared by dissolving the heterocyclic ammonium compound with a calculated excess of HBr in the electrolyte solution.

### 3.3. Electrolyte Formulation

For each BCA, a sample of 10 mL is mixed in aqueous solution containing 5.47 M HBr, 1.11 M [BCA]Br or [BCA]Cl. HBr 48 wt% and H_2_O are added volumetrically. Solubility of [BCA]Br or [BCA]Cl is evaluated. To solutions, in which the BCA salt is dissolved, Br_2_ with a mass of 1.77 g (1.11 M Br_2_) is added gravimetrically. All mixtures correspond to an electrolyte mixture for an SoC of 33%.

For selected BCAs [C2MM]Br, [C2MP]Br, [C2Py]Br, [C4Py]Br, [C6Py]Br, [C2MIm]Br, [C3MIm]Br, [C4MIm]Br and [C6MIm]Br, electrolyte properties are investigated ex situ within the entire SoC range. The SoC range is defined for SoC 0% with 7.7 M HBr, 1.11 M [BCA]Br and 0 M Br_2_ and for an SoC of 100% with 1 M HBr, 1.11 M [BCA]Br and 3.35 M Br_2_. The SoC definition is derived from another study [65]. Samples with a volume of 30 mL in a test series with SoC steps of 10%, and additionally for an SoC of 33% and 66%, are mixed.

### 3.4. Stability of Fused Salt Electrolyte

The stability of the fused salt is checked by visual inspection. The criteria for qualification of the liquid range of a fused salt are the absence of visible orange or red crystals in the fused salt.

### 3.5. Concentration of [BCA]^+^ Cations in Aqueous Electrolytes

Concentrations of 1-alkylpyridin-1-ium cations and 1-alkyl-3-methylimidazol-1-ium cations in the aqueous electrolyte phase are determined by Raman spectroscopy over the entire SoC range. Both cations show characteristic strong peaks at distinctive Raman shifts especially due to their aromaticity [64,75,76]. Characteristic Raman shifts of peaks of the BCAs are shown in Table 2. Peak areas at this Raman shifts are determined and compared with peak areas at known [BCA]^+^ concentrations (an SoC of 0% and 1.11 M [BCA]Br). [BCA]^+^(aq) concentrations as a function of the SoC are therefore calculated (ESI).

### 3.6. Br_2_ Concentration in Aqueous Electrolyte Phase

Br_2_ binding strength of the BCA or solubility of the [BCA]Br_2n+1_ complex is investigated by measurement of Br_2_ concentration in aqueous electrolyte phase. Concentrations of Br_2_ are investigated by linear chronoamperometry at a rotating disk electrode. At a rotation speed of ω = 1000 rpm on vitreous carbon electrode, linear sweep scans between 0.8 V and −0.5 V vs. an Ag/AgCl/KCl(sat.) reference electrode and a scan rate of –40 mV s^−1^ are performed for all samples. Constant reduction currents result for half-cell potentials < −0.1 V vs. Ag/AgCl/KCl(sat.), which represent mass transport limitation by Br_2_ from the bulk phase [77,78]. According to Levich [77,78,79,80], reduction currents are directly proportional to the c(Br_2_) of the bulk solution. Concentrations c(Br_2_(aq)) in M are calculated from the limiting currents I_lim_ (in mA) shown in Equation (6):

c(Br_2_(aq)) = 0.00342466 mol L^−1^ mA^−1^ I_lim_ (6)

A linear calibration curve with known bromine concentrations is investigated before at same conditions. Br_2_ concentrations are measured for the aqueous electrolyte phase of 35 samples with different BCAs chosen at an SoC of 33% and the series of electrolyte samples with [C2Py]Br, [C4Py]Br, [C6Py]Br, [C2MIm]Br, [C3MIm]Br, [C4MIm]Br and [C6MIm]Br at 13 different SoCs.

### 3.7. Polybromide Determination, Polybromide Distribution and [BCA]^+^ Concentration Determination by Raman Analysis

By means of Raman spectroscopy on the aqueous electrolyte samples, second phase crystals and fused salt samples, the occurrence of the different polybromides tribromide Br_3_^−^, pentabromide Br_5_^−^ and heptabromide Br_7_^−^ in electrolyte series of 1-alkylpyridin-1-ium cations und 1-alkyl-3-methylimidazol-1-ium cations electrolytes at different SoCs were investigated. Raman shifts for polybromides and bromine were determined between ṽ = 150 and 320 cm^−1^ as described in the literature [17,56,81,82]. Peak areas of the following stretching oscillations of the polybromides were determined: ṽ(Br_3_^−^, sym.) = 162–163 cm^−1^ [17,53,56], ṽ(Br_3_^−^, antisym.) = 190–198 cm^−1^ [17,56,83], ṽ(Br_5_^−^, antisym.) = 210 cm^−1^ [17,56], ṽ(Br_5_^−^, sym.) = 250–253 cm^−1^ [17,56,81,83,84], ṽ(Br_7_^−^, sym.) = 270 cm^−1^ [56,81]. In the literature, pure Br_2_ shows a strong, single peak at ṽ(Br_2_, sym.) = 300–325 cm^−1^ [81,83,84]. Since there are differences in Raman shifts between the aqueous solution and the corresponding fused salt phase, Raman shifts actually used for fitting the individual peaks are shown in Table 2 for the investigation of the aqueous electrolyte phase. Investigation of Br_2_ distribution in polybromides is explained in detail in the SM and literature [65].

### 3.8. Electrolytic Conductivities of Aqueous Phase

Electrolytic conductivities of the aqueous electrolytes of 1-alkylpyridin-1-ium BCAs and 1-alkyl-3-methylimidazol-1-ium BCAs electrolyte at various SoC are determined at θ = 23 ± 1 °C in a conductivity cell by potentiostatic impedance spectroscopy. The cell constant is determined in a 1 M KCl aqueous solution at θ = 23 ± 1 °C with κ (1 M KCl at θ = 23 °C) = 103.9 mS cm^−^^1^ (calculated from [85]). A detailed description can be found in the ESI.

### 3.9. Redox Potential of the Electrolytes

The redox potentials of the aqueous electrolyte solution are investigated on a glassy carbon stick electrode vs. an Ag/AgCl/KCl(sat.) reference electrode at θ = 23 ± 1 °C and corrected to present them vs. a normal hydrogen electrode. A detailed description can be found in the ESI.

## 4. Conclusions

In this study, tertiary ammonium compounds of pyridine, pyrrolidine, morpholine, 3-methylimidazole, tetraalkylammonium and picolines have been alkylated with *n*-alkyl side chains in the N-position to form organic bromide or chloride salts. In combination with some commercially available organic salts, this resulted in 38 organic quaternary ammonium substances from these six N-containing building blocks. The substances have been investigated ex situ on their properties as BCAs in HBr/Br_2_-electrolytes: BCA solubility, Br_2_ binding strength, the stability of the second heavy fused salt phase, Br_2_ concentration for application, electrolyte conductivity and redox potential. This study has been performed to define their applicability of BCAs to limit the vapor pressure of Br_2_ in aqueous highly concentrated electrolytes for a high-energy density H_2_/Br_2_-RFB with a theoretical capacity of 180 Ah L^−1^/196 Wh L^−1^ (7.7 M HBr).

From the 38 substances evaluated, 15 are not soluble in the electrolyte or form a crystal phase in contact with polybromides. This behavior makes them unsuitable for their application in RFBs. We found that BCAs with short side chains and/or symmetric structure tend to form crystals instead of forming liquid fused salts. An investigation of the [BCA]-polybromide crystal composition showed that all BCA crystals are tribromide salts [BCA]Br_3_.

All the other BCAs form fused salt phases, while most of them exhibit a strong bromine binding strength (14 substances > 95 mol% and four substances between 90 to 95 mol%); four of the N-position protonated BCAs show insufficient binding strengths (<80 mol%). The binding strength increases with an increase in alkyl side chain length, yet the structure of the six basic components (pyridine, picoline, etc.) only has minor influence on the bromine binding strength in solutions.

1-alkyl-3-methylimidazol-1-ium and 1-alkylpyridin-1-ium bromides have been investigated within the whole SoC range to compare the influence of side chain alkyl groups on the different parameters evaluated throughout this study:

For a low SoC (<40%), the selected BCAs show different bromine binding strengths. A clear trend has been found as short alkyl side chains lead to lower Br_2_ binding strength compared to those with long alkyl side chains. For an SoC of ≥40%, the concentrations of Br_2_ in the electrolyte’s aqueous phase increase, while the binding strength becomes independent of the alkylated side chain. However, to obtain a clear insight on the reason behind this behavior, further investigation on the fused salt phase would be needed which is beyond the scope of this study. On a final note regarding this comparison, it is worth mentioning that the ability to form a fused salt phase differed from that of the [C2Py]Br and the [C2MIm]Br BCAs as we observed that while [C2Py]Br forms a liquid fused salt, the cation of [C2MIm]Br tends to crystallize over a wide SoC range.

The ionic conductivity of the aqueous phase is high for the whole SoC range between 325 and 745 mS cm^−1^ and depends primarily on the available proton concentration and its ionic transport by the Grotthus mechanism. The concentration of [BCA]^+^ in the aqueous phase can, to a lesser extent, influence the measured ionic conductivities of the electrolytes as their presence can slightly reduce the conductivity or increase it indirectly by sequestration of Br_2_ from the aqueous phase.

The redox potential of the electrolyte is reduced by 32 to 114 mV due to the use of BCAs compared to BCA-free electrolytes in the whole SoC range. Following the Nernst equation, the decrease in Br_2_ concentration in the aqueous phase leads to reduced redox potentials of the half-cell reaction. This is certainly a trade-off that must be accepted as it is a direct consequence of the ambition to lower bromine vapor pressure by limiting Br_2_ concentration in aqueous solutions.

Based on the results of fused salt stability over the SOC range, electrolyte conductivity, the trend of the redox potential and especially the concentrations of Br_2_ remaining in the aqueous electrolyte phase, one BCA was selected from 38 BCAs and needs to be investigated in detail and applied in the positive half cell of the H_2_/Br_2_-RFB cell: 1-ethylpyridin-1-ium bromide ([C2Py]Br).

## Figures and Tables

**Table 1:** Quaternary ammonium halides for investigation of their properties as bromine complexing agents represented by their structure, name and abbreviation (bold). Letters in brackets identify substances that are synthesized at Fraunhofer ICT **(S)** and are used from commercial suppliers **(C)**. Details are mentioned in the Supplementary Materials (SM).

Quaternary Ammonium Compounds Used as BCAs					
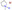	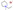	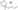	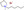	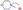	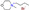
1-Methylpyrrolidin-1-ium hydrobromide, **[HMP]Br (S)**	1-Ethyl-1-methylpyrrolidin-1-iumbromide, **[C2MP]Br (=[MEP]Br) (C)**	1-*n*-Butyl-1-methylpyrrolidin-1-iumbromide, **[C4MP]Br (C)**	1-*n*-Hexyl-1-methylpyrrolidin-1-iumbromide, **[C6MP]Br (S)**	1-Ethyl-1-methylmorpholin-1-iumbromide, **[C2MM]Br (=[MEM]Br) (C)**	1-*n*-Butyl-1-methylmorpholin-1-iumbromide, **[C4MM]Br (C)**
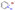	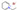	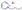	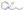	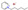	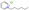
Pyridin-1-ium hydrobromide, **[HPy]Br (S)**	1-Ethylpyridin-1- iumbromide, **[C2Py]Br (S)**	1-*n*-Butylpyridin-1- iumbromide, **[C4Py]Br (S)**	1-*n*-Butylpyridin-1- iumchloride, **[C4Py]Cl (C)**	1-*n*-Hexylpyridin-1-iumbromide, **[C6Py]Br (S)**	1-*n*-Hexylpyridin-1-iumchloride, **[C6Py]Cl (S)**
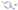	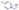	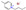	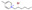	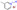	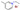
4-Methylpyridine hydrobromide, **[H4MPy]Br (S)**	1-Ethyl-4-methylpyridine hydrobromide, **[C24MPy]Br (S)**	1-*n*-Butyl-4-methylpyridine hydrobromide, **[C44MPy]Br (S)**	1-*n*-Hexyl-4-methylpyridine hydrobromide, **[C64MPy]Br (S)**	3-Methylpyridine hydrobromide, **[H3MPy]Br (S)**	1-Ethyl-3-methylpyridinebromide, **[C23MPy]Br (S)**
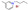	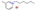	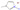	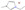	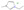	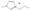
1-*n*-Butyl-3-methyl-pyridinebromide, **[C43MPy]Br (S)**	1-*n*-Hexyl-3-methyl-pyridinebromide, **[C63MPy]Br (S)**	3-Methylimidazol-1-ium hydrobromide, **[HMIm]Br (S)**	1-Ethyl-3-methylimidazol-1-iumbromide, **[C2MIm]Br (S)**	1-Ethyl-3-methylimidazol-1-iumchloride, **[C2MIm]Cl (S)**	1-*n*-Propyl -3-methylimidazol-1-iumbromide, **[C3MIm]Br (S)**
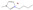	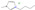	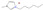	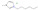	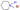	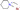
1-*n*-Butyl-3-methyl-imidazol-1-iumbromide, **[C4MIm]Br (S)**	1-*n*-Butyl-3-methyl-imidazol-1-iumchloride, **[C4MIm]Cl (C)**	1-*n*-Hexyl-3-methylimidazol-1-iumbromide, **[C6MIm]Br (S)**	1-*n*-Hexyl-3-methylimidazol-1-iumchloride, **[C6MIm]Cl (S)**	1-Methylpiperidin hydrobromide, **[HMPip]Br (S)**	1-Ethyl-1-methylpiperidinbromide, **[C2MPip]Br (S)**
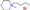	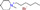	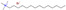		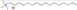	
1-*n*-Butyl-1-methylpiperidinbromide, **[C4MPip]Br (S)**	1-*n*-Hexyl-1-methylpiperidinbromide, **[C6MPip]Br (S)**	1,1,1-Trimethyl-1-*n*-tetradecylammoniumbromide, **[MTA]Br (C)**		1,1,1-Trimethyl-1-*n*-hexadecylammoniumbromide, **[CTA]Br (C)**	
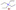	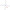	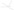		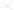	
Tetraethylammoniumbromide, **[TEA]Br (C)**	Tetra-*n*-butylammoniumbromide, **[TBA]Br (C)**	Tetra-*n*-octylammoniumbromide, **[TOA]Br (C)**		Tetra-*n*-octylammoniumchloride, **[TOA]Cl (C)**	

**Table 2:** Selected BCAs to be investigated over the entire SoC range including the zone in which the second phase is present as a liquid fused salt. Furthermore, characteristic Raman shifts of the [BCA]^+^ cation are shown, whereby the Raman peak with the Raman shift in bold is used to determine the [BCA]^+^ concentration. In addition, the Raman shifts of the symmetrical stretching vibration of the individual polybromides are shown, which are used for the evaluation in Section 2.5.

BCA Structure/Name/Abbreviation	SoC Range of Liquid Fused Salt	Characteristic Cation Raman Shifts/cm^−1^	Polybromide Raman Shifts in Aqueous Solutions/cm^−1^	
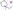	SoC ≥ 30%	899, 1452, 2954, 2964, **2989**	ṽ_S_ (Br_3_^−^)	165–169
			ṽ_S_ (Br_5_^−^)	255
1-Ethyl-1-methylpyrrolidin-1-iumbromide, **[C2MP]Br (= [MEP]Br)**			ṽ_S_ (Br_7_^−^)	269
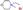	SoC ≥ 60%	685, 701, 2888, 2946, **2985**	ṽ_S_ (Br_3_^−^)	165–167
			ṽ_S_ (Br_5_^−^)	254
1-Ethyl-1-methylmorpholin-1-iumbromide, **[C2MM]Br (= [MEM]Br)**			ṽ_S_ (Br_7_^−^)	269
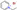	SoC 0–100%	647, **1029**, 1177, 2883, 2919, 2945, 2988, 3098	ṽ_S_ (Br_3_^−^)	165–167
			ṽ_S_ (Br_5_^−^)	253–254
1-Ethylpyridin-1-iumbromide, **[C2Py]Br**			ṽ_S_ (Br_7_^−^)	269
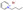	SoC 0–100%	651, **1030**, 1179, 2880, 2914, 2943, 2976, 3097	ṽ_S_ (Br_3_^−^)	167–170
			ṽ_S_ (Br_5_^−^)	254–257
1-*n*-Butylpyridin-1-iumbromide, **[C4Py]Br**			ṽ_S_ (Br_7_^−^)	269
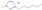	SoC 0–100%	649, **1029**, 2869, 2910, 2939, 2973, 3097	ṽ_S_ (Br_3_^−^)	164–169
			ṽ_S_ (Br_5_^−^)	255–256
1-*n*-Hexylpyridin-1-iumbromide, **[C6Py]Br**			ṽ_S_ (Br_7_^−^)	269
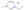	SoC ≥ 50%	598, 1024, 1339, **1419**, 2961	ṽ_S_ (Br_3_^−^)	165–168
			ṽ_S_ (Br_5_^−^)	253–254
1-Ethyl-3-methylimidazol-1-iumbromide, **[C2MIm]Br**			ṽ_S_ (Br_7_^−^)	269
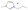	SoC 0–100%	1023, 1337, **1417**, 2961	ṽ_S_ (Br_3_^−^)	167–170
			ṽ_S_ (Br_5_^−^)	254
1-*n*-Propyl -3-methylimidazol-1-iumbromide, **[C3MIm]Br**			ṽ_S_ (Br_7_^−^)	269
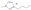	SoC 0–100%	1024, 1339, **1419**, 2963	ṽ_S_ (Br_3_^−^)	165–170
			ṽ_S_ (Br_5_^−^)	254–255
1-*n*-Butyl-3-methylimidazol-1-iumbromide, **[C4MIm]Br**			ṽ_S_ (Br_7_^−^)	269
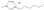	SoC 0–100%	1025, 1122, 1341, **1418**, 2961	ṽ_S_ (Br_3_^−^)	164–167
			ṽ_S_ (Br_5_^−^)	253
1-*n*-Hexyl-3-methylimidazol-1-iumbromide, **[C6MIm]Br**			ṽ_S_ (Br_7_^−^)	269

**Table 3:** Detection limits and detection range for [BCA]^+^ in the aqueous electrolyte phase, maximum fractions of Br_2_ in the aqueous phase compared to the global concentration and c(Br_2_)aq for an SoC of 33%.

[BCA]^+^	[C2Py]^+^	[C4Py]^+^	[C6Py]^+^	[C2MIm]^+^	[C3MIm]^+^	[C4MIm]^+^	[C6MIm]^+^
SoC limit for detectable [BCA]^+^/% ^[a]^	≤70	≤66	≤40	≤60	≤50	≤40	≤33
c(Br_2_)(aq)/c(Br_2_)_total_/% ^[b]^	≤10.1	≤10.3	≤8.5	≤10.4	≤10.0	≤10.6	≤9.5
C(Br_2_)(aq) at an SoC of 30%/mM	121	51	9	105	63	34	8

[a] The SoC value corresponds to the highest SoC at which [BCA]^+^ cations can still be detected in the aqueous phase. [b] Highest ratio of Br_2_ concentration in the aqueous phase versus the total Br_2_ concentration in the sample detected at an SoC of 90 or 100%.

## Data Availability

Data is contained within the article or supplementary material.

## Supplementary Materials

The following are available online: Detailed description of the measurement methods, synthesis of BCAs, manufacturers’ data and chemical purities (Chapter S1), as well as ^1^H NMR and ^13^C NMR interpretation for synthesized BCAs (Chapter S2), tabulation of measured values for Figures within the main manuscript (Table S1–S8), Raman spectra of solutions (Figures S3–S9), Raman spectra of [BCA]Br_3_ crystals (Figure S2) and concentrations of Br_2_ in aqueous solution for all 35 BCAs investigated (Figure S1).
