# Supplementary material for: Systematic Study of Quaternary Ammonium Cations for Bromine Sequestering Application in High Energy Density Electrolytes for Hydrogen Bromine Redox Flow Batteries
Source: Molecules. 2021 May 6;26(9):2721. doi: 10.3390/molecules26092721 (PMC8124678; doi:10.3390/molecules26092721)
Supplement: Supplementary file 1 [file molecules-26-02721-s001.zip › molecules-1143701-supplementary.pdf]

*molecules*

*Supplementary Materials*

# Systematic Study of Quaternary Ammonium Cations for Bromine Sequestering Application in High Energy Density Electrolytes for Hydrogen Bromine Redox Flow Batteries

**Michael Küttinger [1,2], Paulette A. Loichet Torres [1], Emeline Meyer [1], Peter Fischer [1,*] and Jens Tübke [1,2]**

1 Applied Electrochemistry, Fraunhofer Institute for Chemical Technology, Joseph-von-Fraunhofer Straße 7, D-76327 Pfinztal, Germany; michael.kuettinger@gmx.de (M.K.); paulette.loichet@tum.de (P.L.); la-emeline-54@hotmail.fr (E.M.); peter.fischer@ict.fraunhofer.de (P.F.); jens.tuebke@ict.fraunhofer.de (J.T.)

2 Institute for Mechanical Process Engineering and Mechanics, Karlsruhe Institute of Technology KIT, Straße am Forum 8, D-76131 Karlsruhe, Germany; michael.kuettinger@gmx.de (M.K.); jens.tuebke@ict.fraunhofer.de (J.T.)

* Correspondence: Peter Fischer; peter.fischer@ict.fraunhofer.de

**Citation:** Küttinger, M.; Loichet Torres, P.A.; Meyer, E.; Fischer, P.; Tübke, J.; Systematic Study of Quaternary Ammonium Cations for Bromine Sequestering Application in High Energy Density Electrolytes for Hydrogen Bromine Redox Flow Batteries. *Molecules* **2021**, *26*, 2721. https://doi.org/10.3390/molecules26092721

Received: 26 February 2021
Accepted: 26 April 2021
Published: date

**Publisher's Note:** MDPI stays neutral with regard to jurisdictional claims in published maps and institutional affiliations.

**Copyright:** © 2021 by the authors. Licensee MDPI, Basel, Switzerland. This article is an open access article distributed under the terms and conditions of the Creative Commons Attribution (CC BY) license (http://creativecommons.org/licenses/by/4.0/).

In this Supplementary Materials document, the reader can find detailed information on the applied measurement methods, parameter settings for measurements, chemicals and purities, as well as the evaluation steps. In addition, individual results can be found in the form of graphs and tables in Sections 2 to 5 of the ESI. These data supplement the manuscript and ensure the completeness of the work as not all measurement results can be included in the manuscript for reasons of clarity.

## 1. Detailed Description of Experiments and Methods

In addition to the experimental section of the manuscript, the experiments and methods are described in detail in this section of the ESI in order to enable reproducibility of the results. The experiments and evaluation methods are described in detail. The utilized chemicals are named including their manufacturer and purity. For other materials, the names and manufacturers are given.

### *1.1. Reagents*

The reagents used for the preparation of bromine electrolyte samples in this work were the following: hydrobromic acid (48 % w/w, Alpha Aesar), bromine (≥ 99 %, Sigma Aldrich), distilled water (PURELAB Ultra Analytic/Veolia-ELGA). For the synthesis of BCAs, the following chemicals were utilized: 1-methylimidazole (99 %, Aldrich), pyridine (99 %, Alpha Aesar), 1-methylpiperidine (99 %, Alpha Aesar); 1methylpyrrolidine (98 %, Alpha Aesar), 3-methylpyridine/3-picoline (99 %, Alpha Aesa), 4methylpyridine/4-picoline (98 %, Alpha Aesar), bromoethane (98 %, Alpha Aesar), 1-bromopropane (99 %, Alpha Aesar), 1-bromobutane (98 %+, Alpha Aesar), 1-bromohexane (98 %, Alpha Aesar). The following BCAs were used from commercial suppliers: 1-n-butylpyridin-1-ium chloride [C4Py]Cl (99 %, IOLITEC); 1-n-butyl-3-methylimidazol-1-ium chloride [C4MIm]Cl (99 %, IOLITEC), tetraethylammonium bromide [TEA]Br (≥ 99%, Carl Roth), tetra-n-butylammonium bromide [TBA]Br (≥ 99 %, Carl Roth), tetra-n-octylammonium bromide [TOA]Br (≥ 99 %, Carl Roth), tetra-noctylammonium chloride [TOA]Cl (≥ 99 %, Carl Roth), 1-n-hexadecyltrimetyhlammonium bromide [CTA]Br (≥ 99 %, Carl Roth), *N*,*N*,*N*-Trimethyl-1-tetradecanammoniumbromid [MTA]Br (≥ 96 %, Carl Roth). 1-ethyl-1-methylpyrrolidin-1-ium bromide [C2MP]Br (known in literature as [MEP]Br), 1-butyl1-methylpyrroldin-1-ium bromide [C4MP]Br, 1-ethyl-1-methylmorpholinium bromide [C2MM]Br (known in literature as [MEM]Br) and 1-butyl-1-methylmorpholinium bromide

[C4MM]Br are water diluted solutions with a water content of 20 wt% (Israel Chemicals Limited – Industrial Products (ICL-IP).

*1.2. Synthesis of Bromine Complexing Agents*

The investigated BCAs are listed in Table 1 in the article, including chemical structure, name and abbreviation. Abbreviations are used in the manuscript and the ESI to ease the readability of the manuscript. Non-commercially available N-alkylated quaternary ammonium bromide salts based on pyridine ([C2Py]Br, [C4Py]Br, [C6Py]Br, [C6Py]Cl), on 3-picoline ([C23MPy]Br, [C43MPy]Br, [C63MPy]Br), on 4-picoline ([C24MPy]Br, [C44MPy]Br, [C64MPy]Br), on 1-methylimidazole ([C2MIm]Br, [C3MIm]Br, [C4MIm]Br, [C6MIm]Br, [C6MIm]Cl), on 1-methylpiperidine ([C2MPip]Br, [C4MPip]Br, [C6MPip]Br and on 1-methylpyrrolidine ([C6MP]Br) are synthesized by alkylation reaction [1,2] in a one step and solvent-free nucleophilic substitution reaction ($S_N2$ preferred) according to Dzyuba et al. [3]. Exclusively tertiary amines and bromoalkanes or chloroalkanes participate in the reaction. The reaction is followed by a single-step cleaning process. [C2MIm]Br, [C3MIm]Br, [C4MIm]Br and [C6MIm]Br form a crude golden liquid in the synthesis also described in the literature [3]. For the synthesis of all the other BCAs, the same method and conditions are applied. Some BCAs are available as golden or even reddish liquids at reaction temperatures between 40 °C and 100 °C, while some precipitate as golden or white solids due to their insolubility in their reaction educts. The amounts of the synthesized products are 0.1 mol of [BCA]Br for the first tests of stability and solubility. For the SoC-dependent electrolyte series, 1.5 mol of [BCA]Br is produced. Products are purified in a single-step process by evaporating low-boiling educts and resting in a vacuum chamber at p = 90-150 mbar and $\vartheta$ = 80 °C for at least 24 h. $^1$H NMR and $^{13}$C NMR spectra of the synthesized substances are recorded and confirm the absence of any educts as well as water left in the product. $^1$H NMR and $^{13}$C NMR shifts, as well as instrument data, are presented in the following section of the ESI and are consistent with NMR shifts found in the literature [2,4–7]. Protonated forms of 4-picoline, 3-picoline, 1-methylpiperidine, pyridine, 1methylimidazole and 1-methylpyrrolidine ([H4MPy]Br, [H3MPy]Br, [HMP]Br, [HMPip]Br, [HPy]Br, [HMIm]Br) are prepared by dissolving the heterocyclic ammonium compound with a calculated excess of HBr in the electrolyte solution without any further or prior purification.

*1.3. Electrolyte Formulation*

For each BCA, a 10 mL sample is mixed containing 5.47 M HBr, 1.11 M [BCA]Br or [BCA]Cl. HBr 48 wt%, and $H_2O$ is added volumetrically and the solubility of [BCA]Br or [BCA]Cl in the electrolyte is evaluated. To solutions in which the BCA is completely dissolved, $Br_2$ with a mass of 1.77 g (1.11 M $Br_2$) is added dropwise and gravimetrically. All mixtures correspond to an electrolyte mixture for an SoC of 33 %. Solutions are shaken daily once for two weeks to achieve a chemical equilibrium between the two electrolyte phases. For selected BCAs: [C2MP]Br, [C2Py]Br, [C4Py]Br, [C6Py]Br, [C2MIm]Br, [C3MIm]Br, [C4MIm]Br and [C6MIm]Br, the electrolyte properties are investigated ex situ within the entire SoC range. The SoC range is defined for SoC 0 % with 7.7 M HBr, 1.11 M [BCA]Br and 0 M $Br_2$ and for SoC 100 % with 1 M HBr, 1.11 M [BCA]Br and 3.35 M $Br_2$. The SoC definition is derived from another study dealing with limits in the cycling capacity of pure HBr/$Br_2$/$H_2O$ electrolytes [8]. Samples in a test series with SoC steps of 10 % and additionally for an SoC of 33 % and 66 % are mixed. For an SoC of 100 %, a ratio of n([BCA]$^+$) : n($Br_2$) = 1:3 is obtained by mixing solutions with a volume of 30 mL. The amount of water is kept constant for all samples and is based on a 7.7 M HBr solution defined for an SoC of 0 % to maintain the theoretical amount of water in the posolyte during $H_2$/$Br_2$-RFB cell operation. Electrolytes are shaken daily once for two weeks to achieve a chemical equilibrium between the two electrolyte phases. Electrolyte mixtures

over the SoC range in the 30 mL samples are defined by compound concentrations shown in Table S1:

**Table 1.** Total concentrations of $Br_2$, HBr and [C2Py]Br in mol $L^{-1}$ in the investigated electrolyte samples at chosen SoC for ex situ electrolyte investigation.

| | Total concentrations of Br2, HBr and [C2Py]Br in mol $L^{-1}$ in investigated electrolyte samples at chosen SoC | | | | | | | | | | | | |
|---|---|---|---|---|---|---|---|---|---|---|---|---|---|
| **SoC / %** | **0** | **10** | **20** | **30** | **33** | **40** | **50** | **60** | **66** | **70** | **80** | **90** | **100** |
| c(HBr) / M | 7.70 | 7.03 | 6.36 | 5.69 | 5.49 | 5.02 | 4.35 | 3.68 | 3.28 | 3.01 | 2.34 | 1.67 | 1.00 |
| c([C2Py]Br) / M | 1.11 | 1.11 | 1.11 | 1.11 | 1.11 | 1.11 | 1.11 | 1.11 | 1.11 | 1.11 | 1.11 | 1.11 | 1.11 |
| c(Br2) / M | 0.00 | 0.34 | 0.67 | 1.01 | 1.11 | 1.34 | 1.68 | 2.01 | 2.21 | 2.35 | 2.68 | 3.02 | 3.35 |

### *1.4. Stability of Fused Salt Electrolyte*

Electrolyte samples for different, soluble BCAs at an SoC of 33 % are shaken daily once for two weeks. All electrolytes whose properties are investigated as a function of SoC are also shaken daily once for two weeks. The stability of the fused salt is checked by visual inspection. Criteria for qualification of the liquid range of a fused salt are the absence of visible orange or red crystals formed during these two weeks. By shaking for a period of two weeks, a chemical equilibrium for $Br_2$ and $[BCA]^+$ cations between the two phases is achieved.

### *1.5. Concentration of $[BCA]^+$ Cations in Aqueous Electrolytes*

Concentrations of 1-alkylpyridin-1-ium cations and 1-alkyl-3-methylimidazol-1-ium cations in aqueous electrolyte phase are determined by Raman spectroscopy. Both cations show characteristic strong peaks at distinctive Raman shifts due to their aromaticity in particular. Peaks do not overlap with the Raman shifts of polybromide oscillations ($100\ cm^{-1} \le \tilde{\nu} \le 350\ cm^{-1}$ [9,10]) and oscillations of the water ($\tilde{\nu} = 1600\ cm^{-1}$; $2800\ cm^{-1} \le \tilde{\nu} \le 3800\ cm^{-1}$ [11–14]). Characteristic Raman shifts of peaks of the BCAs are shown in Table 2 in the article. For 1-alkylpyridin-1-ium cations, the peak areas (A) are determined at $\tilde{\nu}$ = 1029-1030 $cm^{-1}$ [15–17], and for 1-alkyl-3-methylimidazol-1-ium cations the peak is at $\tilde{\nu}$ = 1418-1419 $cm^{1}$ [15] according to Lorentz model using the Levenberg–Marquardt iteration algorithm. For an SoC of 0 %, the $[BCA]^+(aq)$ concentrations of 1.11 M are known for the corresponding peak areas. The $[BCA]^+(aq)$ concentrations as a function of the SoC are therefore calculated according to Eqn. S1:

$$1\ c([BCA]^+(aq)) = \frac{A_{1029|1419\ cm^{-1}}{}_{aq}}{A_{1029|1419\ cm^{-1}}{}_{aq\ (SoC\ 0\ \%)}} \cdot 1.11\ mol\ L^{-1} \tag{1}$$

$[c_{[BCA]+aq}] = mol\ L^{-1}$

### *1.6. $Br_2$ Concentration in Aqueous Electrolyte Phase*

$Br_2$ binding strength of BCAs or $Br_2$ solubility is measured by investigation of $Br_2$ concentration in the aqueous electrolyte phase and comparison with $Br_2$ concentration of pure $HBr/Br_2$ electrolytes at the same SoC. Concentrations of $Br_2$ are investigated by linear chronoamperometry on a rotating disk electrode (Gamry RDE 710 Rotating Electrode). At $\omega$ = 1000 rpm on a vitreous carbon electrode (5 mm), (RDE0008, Fixed-Disk Electrode GC RDE Tip PTFE, Ametek, USA) linear sweep excitations between 0.8 V and -0.5 V vs. a silver/silver chloride/potassium chloride reference electrode Ag/AgCl/KCl(sat.) (SE 11/Sensortechnik Meinsberg) and a scan rate of -40 mV $s^{-1}$ were performed for all samples. A platinum counter electrode was applied in the cell. During the reduction process, a diffusion layer of constant thickness was formed by rotation and did not grow into the interior of the solution by forced convection [18–20]. Constant reduction currents resulted for

half-cell potentials < -0.1 V vs. Ag/AgCl/KCl(sat.), which are potential independent in this range and purely represent mass transport limitation by the $Br_2$ from the bulk phase [19,20]. According to Levich [18–21], reduction currents are directly proportional to the concentration c($Br_2$) of the bulk solution represented by Eqn. S2:

$$I_{diff;lim} = 0.62\ FA_{geo}(D_{Br2})^{\frac{2}{3}}\omega^{\frac{1}{2}}\nu^{-\frac{1}{6}}c_{Br_2;aq} \quad (2)$$

$[I_{diff;lim}]$ = mA

Concentrations are calculated from the limiting currents. A linear calibration curve with known bromine concentrations (0.05, 0.1, 0.2, 0.3, 0.4, 0.5 M $Br_2$ in M HBr) is investigated earlier under the same conditions in order to gain the constant $0.62\ FA_{geo}(D_{Br2})^{3}\omega_{2}\nu^{-6}$ of Eqn. S2.

According to Eqn. S3, the concentration c($Br_{2;\ aq}$) is calculated as follows:

$$c(Br_2(aq)) = 0.00342466\ mol\ L^{-1}\ mA^{-1} \cdot I_{diff;\lim} \quad (3)$$

$[c(Br_2(aq))] = mol\ L^{-1}$

$Br_2$ concentrations are measured for the aqueous electrolyte phase of 35 samples with different BCAs chosen at an SoC of 33 % (Figure 2 of the article) and the series of electrolyte samples with [C2Py]Br, [C4Py]Br, [C6Py]Br, [C2MIm]Br, [C3MIm]Br, [C4MIm]Br and [C6MIm]Br at 12 different SoCs (10, 20, 30, 33, 40, 50, 60, 66, 70, 80, 90, 100 %)

### *1.7. Polybromide Determination and $Br_2$ Distribution in Aqueous Phases*

By means of Raman spectroscopy on the aqueous electrolyte samples, the occurrence of the different polybromides tribromide $Br_3^-$, pentabromide $Br_5^-$ and heptabromide $Br_7^-$ in the electrolyte series of 1alkylpyridin-1-ium cations and 1-alkyl-3-methylimidazol-1-ium cations electrolytes at different SoCs (10, 20, 30, 33, 40, 50, 60, 66, 70, 80, 90, 100 %) are investigated. Raman spectra are measured with the HORIBA LabRAM HR spectrometer (JOBIN YVON Technology GmbH, Germany) at room temperature $\vartheta$ = 23 ± 1 C in the wavenumber ($\tilde{\nu}$) range from $\tilde{\nu}$ = 50 $cm^{-1}$ to 4000 $cm^{-1}$. A frequency-doubled Nd:YAG laser (neodymium-doped yttrium–aluminum–garnet laser) with an excitation wavelength of $\lambda$ = 532 nm (green) is used as laser source and a charge-coupled device camera detector (CCD) at $\vartheta$ = -70 °C. Aqueous electrolyte samples are pipetted into a quartz glass cuvette (Suprasil QS, Helma), sealed and examined under the microscope. The focal point is positioned just below the edge of the front glass of the cuvette and the position of the carrier table is fixed to obtain reproducible Raman spectra. For all samples and wavenumbers, the exposure time was 10 s and the number of repetitions per sample for calculating the average was set to three.

Raman shifts for polybromides and bromine were determined for Raman shifts between $\tilde{\nu}$ = 150 and 320 $cm^{-1}$ as described in the literature. [9,10,22,23]. Obtained Raman spectra were corrected in the Rayleigh range, and the areas of the following stretching oscillations of the polybromides were determined by fitting according to the Lorentz model and using the iteration algorithm according to Levenberg–Marquardt: $\tilde{\nu}$($Br_3^-$,sym.) ≈ 164-170 $cm^{-1}$ [10,22,24], $\tilde{\nu}$($Br_3^-$,antisym.) ≈ 190-198 $cm^{-1}$ [10,22,25], $\tilde{\nu}$($Br_5^-$,antisym.) ≈ 210 $cm^{1}$ [10,22], $\tilde{\nu}$($Br_5^-$,sym.) ≈ 253-255 $cm^{-1}$ [10,22,23,25,26], $\tilde{\nu}$($Br_7^-$,sym.) ≈ 269 $cm^{-1}$ [10,23]. In the literature, pure $Br_2$ shows a strong single peak at $\tilde{\nu}$($Br_2$,sym.) ≈ 300-325 $cm^{-1}$ [23,25,26]. Since there are differences in Raman shifts between the aqueous solution and the corresponding fused salt phase, the Raman shifts actually used for the individual peaks are shown in Table 2 of the article. Stretching vibrations in polybromides occur between the inner bromide ion and the outer atoms of the $Br_2$ molecule attached via an addition bonding [8,10]. Due to the addition of two $Br_2$ molecules, $Br_5^-$ twice the intensity of a $Br_3^-$ anion can be expected. For $Br_7^-$, the intensity is expected to be three times as high as that of $Br_3^-$ anions. The areas under the curves of the symmetric stretching oscillation correspond to the sum of the $Br_2$ molecules involved in this stretching vibration of one polybromide type. The fraction of the area of a symmetrical stretching oscillation in relation to the sum of all

areas of the symmetrical stretching oscillations of $Br_3^-$, $Br_5^-$ and $Br_7^-$ gives the fraction of the $Br_2$ concentration present in each polybromide. A detailed description and explanation of the method is printed in [8]. All Raman spectra of the aqueous phase are shown in Figure S3–S9 in this ESI.

### *1.8. Electrolytic Conductivities of Aqueous Phase Posolyte*

Electrolytic conductivities of the aqueous electrolytes with 1-alkylpyridin-1-ium BCAs and 1-alkyl-3methylimidazol-1-ium BCAs electrolyte mixtures at various SoC (0, 10, 20, 30, 33, 40, 50, 60, 66, 70, 80, 90 and 100 %) are determined at $\vartheta = 23 \pm 1$ °C. In a conductivity cell LF 1101 (SI Analytics GmbH/Germany), ohmic electrolyte resistances $R_{Electrolyte,\ measured}$ are measured by potentiostatic impedance spectroscopy with a perturbation of û = 10 mV (amplitude) in the range of 1 MHz to 100 Hz, without potential offset. Ohmic resistances $R_{Electrolyte,\ measured}$ from the measurement are corrected by the ohmic resistance of cables and connections to gain $R_{Electrolyte}$. Cell constants $K_{Conductometer}$ are determined for each row of 13 measurements in a 1 M KCl solution at $\vartheta = 23$ °C. The conductivity of 1 M KCl solution at $\vartheta = 23$ °C is determined by linear regression from the literature [27] to be $\kappa_{1M\ KCl, \vartheta=23°C} = 103.9$ mS $cm^{-1}$. The sensor is rinsed first with acetone and then twice with purified water. $K_{Conductometer}$ and electrolyte conductivity $\kappa_{Electrolyte}$ are calculated by means of Eqn. S4 and Eqn. S5:

$$K_{Conductometer} = \frac{A}{l} = \frac{1}{R_{1M\ KCl,\ \vartheta=23°C} \cdot \kappa_{1M\ KCl,\ \vartheta=23°C}} \quad (4)$$

[K] = cm

$$\kappa_{Elektrolyt,\ t=23°C} = \frac{1}{R_{Electrolyte,\ \vartheta=23°C} \cdot K_{Conductometer}} \quad (5)$$

[κ] = S $cm^{-1}$

### *1.9. Redox Potential of the Electrolytes*

Redox potentials of the aqueous electrolyte solution are investigated on a glassy carbon stick electrode vs. a silver/silver chloride/potassium chloride reference electrode Ag/AgCl/KCl(sat.) at $\vartheta = 23 \pm 1$ °C with $\varphi_{Ag/AgCl/KCl(sat.)} = 198$ mV [19] vs. normal hydrogen electrode (NHE). Both electrodes are dipped into the aqueous solution, and the redox potential is measured when the potential becomes stable. After this, the redox potential is corrected to show the redox potential vs. NHE in this work.

## 2. $^1$H NMR and $^{13}$C NMR Characterization of Synthesized Bromine Complexation agents

$^1$H NMR and $^{13}$C NMR spectra are recorded as shown below and the synthesis of the expected BCAs is confirmed by the existing literature [2,4–7,28,29]. $^1$H NMR and $^{13}$C NMR spectra are measured on a 400 MHz Bruker AV-400 spectrometer (400 MHz for $^1$H NMR and 100 MHz for $^{13}$C NMR) using DMSO-$d_6$ as a solvent. The $^1$H NMR data are reported in ppm (δ) from the internal standard (TMS, 0.0 ppm) and chemical shift (multiplicity, coupling constant in Hz, integration), and the $^{13}$C NMR data are in ppm (δ) from the internal standard (TMS, 0.0 ppm). NMR spectra are only examined for the chemicals listed in Table 1 of the manuscript, which were not purchased commercially. These chemicals are listed in the experimental section in the sub-section "Synthesis of bromine complexing agents" of the manuscript:

1-ethylpyridin-1-ium bromide [C2Py]Br

$^1$H NMR (400 MHz, DMSO-$d_6$) δ(ppm): 1.53 (t, J = 7.2 Hz, 3H), 4.69 (q; J = 7.3 Hz, 2H), 8.18 (t, J = 7.1 Hz, 2H), 8.62 (t, J = 7.8 Hz, 1H), 9.10-9.53 (m, 2H). $^{13}$C NMR (100 MHz, DMSO-$d_6$) δ(ppm): 16.38, 56.19, 128.02, 144.55, 145.39.

1-*n*-butylpyridin-1-ium bromide [C4Py]Br

$^{1}$H NMR (400 MHz, DMSO-d$_6$) δ(ppm): 0.89 (t, J = 7.2 Hz, 3H), 1.16-1.33 (m, 2H), 1.74-1.94 (m, 2H), 4.58 (t; J = 7.4 Hz, 2H), 8.15 (t, J = 7.1 Hz, 2H), 8.63 (t, J = 7.8 Hz, 1H), 9.12-9.47 (m, 2H). $^{13}$C NMR (100 MHz, DMSO-d$_6$) δ(ppm): 13.31, 18.69, 32.67, 60.33, 128,04, 144,76, 145.

1-*n*-hexylpyridinium bromide [C6Py]Br

$^{1}$H NMR (400 MHz, DMSO-d$_6$) δ(ppm): 0.83 (t, J = 7.5 Hz, 3H), 1.12-1.37 (m, 6H), 1.80-1.98 (m, 2H), 4.57 (t; J = 7.6 Hz, 2H), 8.20 (t, J = 7.0 Hz, 2H), 8.60 (t, J = 7.7 Hz, 1H), 9.05-9.40 (m, 2H). $^{13}$C NMR (100 MHz, DMSO-d$_6$) δ(ppm): 13.77, 21.80, 24.97, 30.50, 30.68, 60.54, 128.04, 144.75, 145.47.

1-ethyl-3-methylimidazol-1-ium bromide [C2MIm]Br

$^{1}$H NMR (400 MHz, DMSO-d$_6$) δ(ppm): 1.40 (t, J = 7.3 Hz, 3H), 3.86 (s; 3H), 4.21 (q, J = 7.3 Hz, 2H), 7.81 (t, J = 1.9 Hz, 2H), 9.31 (s, 1H). $^{13}$C NMR (100 MHz, DMSO-d$_6$) δ(ppm): 15.14, 35.70, 44.06, 121.93, 123.48, 136.24.

1-*n*-propyl-3-methylimidazol-1-ium bromide [C3MIm]Br

$^{1}$H NMR (400 MHz, DMSO-d$_6$) δ(ppm): 0.84 (t, J = 7.5Hz, 3H), 1.71-1.86 (m, 2H), 3.87 (s; 3H), 4.15 (t, J = 7.1 Hz, 2H), 7.79 (t, J = 1.6 Hz, 2H), 9.30 (s, 1H). $^{13}$C NMR (100 MHz, DMSO-d$_6$) δ(ppm): 10.38, 22.83, 35.73, 50.16, 122.23, 123.54, 136.5.

1-*n*-butyl-3-methylimidazol-1-ium bromide [C4MIm]Br

$^{1}$H NMR (400 MHz, DMSO-d$_6$) δ(ppm): 0.87 (t, J = 7.4 Hz, 3H), 1.21-1.37 (m, 2H), 1.70-1.88 (m, 2H), 3.86 (s, 3H), 4.18 (t; J = 7.3 Hz, 2H), 7.65 (t, J = 1.8 Hz, 2H), 9.15 (s, 1H). $^{13}$C NMR (100 MHz, DMSO-d$_6$) δ(ppm): 13.25, 18.72, 31.34, 35.74, 48.40, 122.23, 123.55, 136.50.

1-*n*-hexyl-3-methylimidazol-1-ium bromide [C6MIm]Br

$^{1}$H NMR (400 MHz, DMSO-d$_6$) δ(ppm): 0.78-0.93 (m, 3H), 1.18-1.39 (m, 6H), 1.71-1.85 (m, 2H), 3.86 (s, 3H), 4.23 (t, J = 7.21 Hz, 2H), 7.41 (t, J = 1.7 Hz, 2H) 9.26 (s, 1H). $^{13}$C NMR (100 MHz, DMSO-d$_6$) δ(ppm): 13.80, 21.84, 25.09, 29.33, 30.51, 35.74, 48.68, 122.22, 123,53, 136,49.

1-ethyl-4-methylpyridin-1-ium bromide [C24MPy]Br

$^{1}$H NMR (400 MHz, DMSO-d$_6$) δ(ppm): 1.50 (t, J = 7.3 Hz, 3H), 2.60 (s, 3H), 4.60 (q, J = 7.3 Hz, 2H), 8.00 (d, J = 6.57 Hz, 2H), 9.04 (d, J = 6.57 Hz, 2H). $^{13}$C NMR (100 MHz, DMSO-d$_6$) δ(ppm): 16.29, 21.31, 55.34, 128.29, 143.51, 158.58.

1-*n*-butyl-4-methylpyridin-1-ium bromide [C44MPy]Br

$^{1}$H NMR (400 MHz, DMSO-d$_6$) δ(ppm): 0.89 (t, J = 7.3 Hz, 3H), 1.16-1.33 (m, 2H), 1.74-1.94 (m, 2H), 2.60 (s, 3H), 4.58 (t; J = 7.5 2H), 8.01 (d, J = 6.3 Hz, 2H), 9.03 (d, J = 6.8 Hz, 2H). $^{13}$C NMR (100 MHz, DMSO-d$_6$) δ(ppm): ): 13.29, 18.66, 21.33, 32.53, 59.50, 128.30, 143.72, 158.70.

1-*n*-hexyl-4-methylpyridin-1-ium bromide [C64MPy]Br

$^{1}$H NMR (400 MHz, DMSO-d$_6$) δ(ppm): 0.76-0.92 (m, 3H), 1.11-1.36 (m, 6H), 1.79-1.95 (m, 2H), 2.60 (s, 3H), 4.58 (t; J = 7.5, 2H), 8.05 (d, J = 6.3 Hz, 2H), 9.05 (d, J = 6.8 Hz, 2H). $^{13}$C NMR (100 MHz, DMSO-d$_6$) δ(ppm): 13.78, 21.33, 21.82, 24.96, 30.51, 30.54, 59.71, 128.29, 143.71, 158.68.

1-ethyl-3-methylpyridin-1-ium bromide [C23MPy]Br

$^{1}$H NMR (400 MHz, DMSO-d$_6$) δ(ppm): 1.53 (t, J = 7.3 Hz, 3H), 2.51 (s, 3 H), 4.63 (q, J = 7.3 Hz, 2H), 8.07 (dd, J = 7.8, 6.3 Hz, 1H), 8.46 (d, J = 8.1 Hz, 1 H), 9.04 (d, J = 6.1 Hz, 1 H) 9.17 (s, 1H). $^{13}$C NMR (100 MHz, DMSO-d$_6$) δ(ppm): 16.32, 17.81, 56.01, 127.30, 138.60, 141.73, 144.10, 145.62.

1-*n*-butyl-3-methylpyridin-1-ium bromide [C43MPy]Br

$^{1}$H NMR (400 MHz, DMSO-d$_6$) δ(ppm): 0.89 (t, J = 7.3 Hz, 3H), 1.18-1.35 (m, 2H), 1.80-1.91 (m, 2H), 2.49 (s, 3 H), 4.59 (t, J = 7.5 Hz, 2H), 8.07 (dd, J = 8.0, 6.2 Hz, 1H), 8.46 (d, J = 8.1 Hz, 1 H), 9.01 (d, J = 6.1 Hz, 1 H), 9.13 (s, 1H). $^{13}$C NMR (100 MHz, DMSO-d$_6$) δ(ppm): 13.30, 17.82, 18.72, 32.57, 60.21, 127.29, 138.68, 141.96, 144.25, 145.71.

1-*n*-hexyl-3-methylpyridin-1-ium bromide [C63MPy]Br

$^{1}$H NMR (400 MHz, DMSO-d$_6$) δ(ppm): 0.73-0.93 (m, 3H), 1.11-1.36 (m, 6H), 1.90 (t, J = 7.1 Hz, 2H), 2.49 (s, 3 H), 4.57 (t, J = 7.6 Hz, 2H), 8.06 (dd, J = 7.8, 6.1 Hz, 1H), 8.45 (d, J =

7.83 Hz, 1 H), 9.00 (d, J = 5.8 Hz, 1 H), 9.11 (s, 1H). $^{13}C$ NMR (100 MHz, DMSO-d$_6$) δ(ppm): 13.79, 17.82, 21.80, 25.00, 30.51, 30.57, 60.44, 127.28, 138.67, 141.95, 144.24, 145.71.

1-*n*-hexyl-1-methylpyrrolidin-1-ium bromide [C6MP]Br

$^1H$ NMR (400 MHz, DMSO-d$_6$) δ(ppm): 0.81-0.92 (m, 3H), 1.19-1.37 (m, 6H), 1.68 (dt, J = 7.89, 4.01 Hz, 2H), 2.07 (m, 4 H), 3.00 (s, 3H), 3.28-3.38 (m, 2H), 3.39-3,58 (m, 4 H). $^{13}C$ NMR (100 MHz, DMSO-d$_6$) δ(ppm): 13.81, 21.02, 21.85, 22.86, 25.33, 30.64, 47.42, 62.89, 63.29.

1-ethyl-1-methylpiperidin-1-ium bromide [C2MPip]Br

$^1H$ NMR (400 MHz, DMSO-d$_6$) δ(ppm): 0.95 (t, J = 6.5 Hz, 3H), 1.25-1.34 (m, 2H), 2.00-2.15 (m, 4 H), 3.01 (s, 3H), 3.30 (t, J = 6.4 Hz, 2H), 3.40-3.59 (m, 4H). $^{13}C$ NMR (100 MHz, DMSO-d$_6$) δ(ppm): 13.50, 19.27, 21.01, 47.41, 62.66, 63.28.

1-*n*-butyl-1-methylpiperidin-1-ium bromide [C4MPip]Br

$^1H$ NMR (400 MHz, DMSO-d$_6$) δ(ppm): 0.91 (t, J = 6.5 Hz, 3H), 1.25-1.34 (m, 2H), 1.51-1.68 (m, 4H), 1.72-1.81 (m, 4 H), 3.01 (s, 3H), 3.25-3.41 (m, 4H). $^{13}C$ NMR (100 MHz, DMSO-d$_6$) δ(ppm):13.53, 19.27, 20.65, 20.76, 22.95 46.94, 59.79, 62.02.

1-*n*-hexyl-1-methylpiperidin-1-ium bromide [C6MPip]Br

$^1H$ NMR (400 MHz, DMSO-d$_6$) δ(ppm): 0.92 (t, J = 6.4 Hz, 3H), 1.20-1.38 (m, 6H), 1.55 (m, 2H), 1.79 (m, 4 H), 2.48 (t, J = 6.8 Hz, 2H), 3.03 (s, 3H), 3.25-3.40 (m, 4H). $^{13}C$ NMR (100 MHz, DMSO-d$_6$) δ(ppm): 13.80, 19.26, 20.65, 20.88, 21.86, 25.48, 30.66, 46.91, 59.77, 62.23.

## 3. Additional Figures in Connection with the Main Manuscript

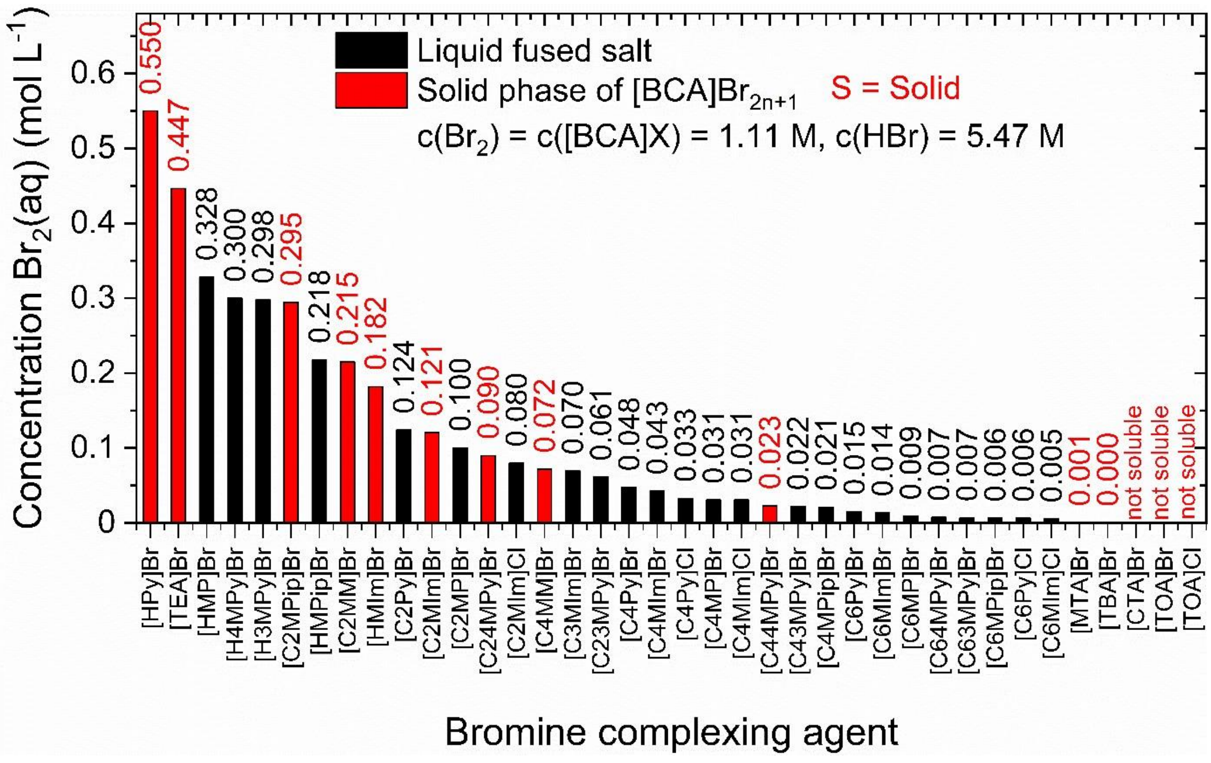


**Figure 1.** Concentrations of $Br_2$ in aqueous electrolyte solutions containing different BCAs (5.47 M HBr, 1.11 M $Br_2$ and 1.11 M [BCA]Br or [BCA]Cl in water). Concentration values are shown above the bars in mol $L^{-1}$. Red bars show concentrations of bromine in the aqueous phase for electrolytes with crystalline second phase and black bars for the aqueous phase of electrolytes with liquid fused salt.

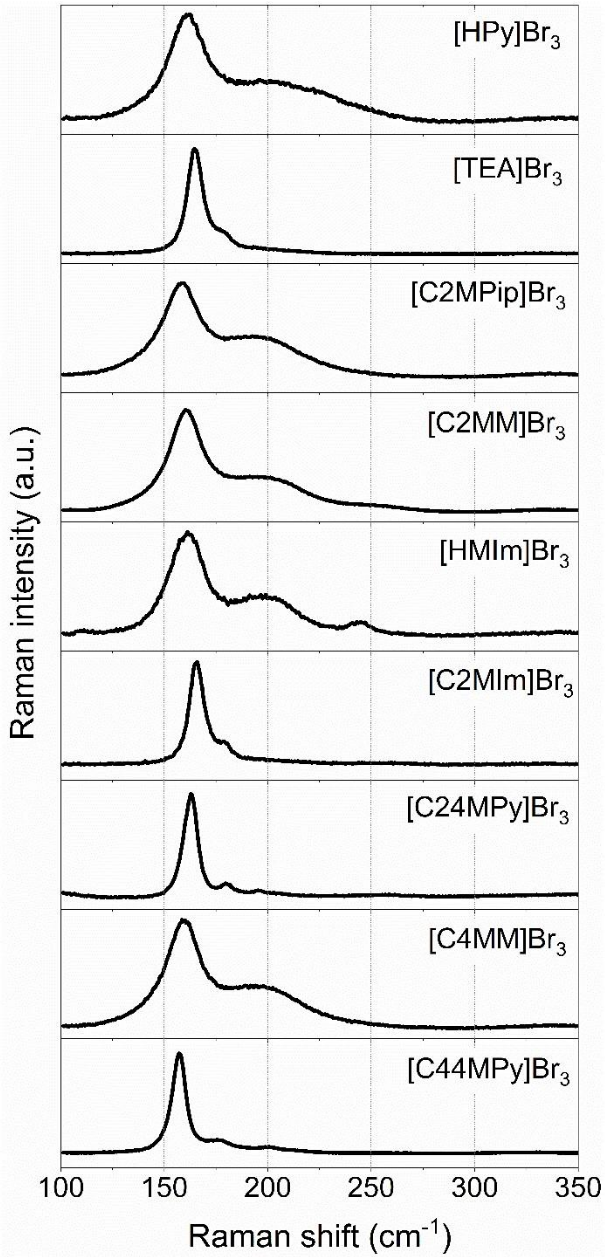


**Figure 2.** Raman spectra of all crystals formed by mixing 5.47 M HBr, 1.11 M $Br_2$ and 1.11 M [BCA]Br. All Raman spectra show in the Raman shift range between 150 $cm^{-1}$ < $\tilde{\nu}$ <200 $cm^{-1}$ peaks of symmetrical and antisymmetrical stretching oscillation of tribromide $Br_3^-$ anions in the [BCA]$Br_3$ crystals. Higher polybromides $Br_5^-$ and $Br_7^-$ are not available in the dried crystals as there symmetric stretching oscillation would be expected between 210 $cm^{-1}$ < $\tilde{\nu}$ <300 $cm^{-1}$. For crystals of [MTA]Br and [TBA]Br as BCA, no Raman spectra were investigated. For [HMIm]$Br_3$, crystals were wet during measurement and show a small Raman peak at $\tilde{\nu}$ ≈ 245 $cm^{-1}$. It was not possible to dry them further without decomposition.

## 4. Results of the Manuscript in Tabulated Form

**Table 1.** Concentration of $[BCA]^+$ cations in aqueous electrolyte solutions depending on SoC (mixture of the electrolyte) in mol $L_{-1}$.

| SoC (%) | [C2Py]Br (aq) | [C4Py]Br (aq) | [C6Py]Br (aq) | [C2MIm]Br (aq) | [C3MIm]Br (aq) | [C4MIm]Br (aq) | [C6MIm]Br (aq) |
|---|---|---|---|---|---|---|---|
| 0 | 1.110 | 1.110 | 1.110 | 1.110 | 1.110 | 1.110 | 1.110 |
| 10 | 0.914 | 0.877 | 0.921 | 0.792 | 0.807 | 0.795 | 0.914 |
| 20 | 0.697 | 0.566 | 0.471 | 0.544 | 0.581 | 0.521 | 0.566 |
| 30 | 0.447 | 0.237 | 0.144 | 0.283 | 0.320 | 0.281 | 0.128 |
| 33 | 0.307 | 0.267 | 0.119 | 0.201 | 0.259 | 0.187 | 0.148 |
| 40 | 0.175 | 0.094 | 0.030 | 0.174 | 0.148 | 0.073 | 0 |
| 50 | 0.132 | 0.044 | 0 | 0.131 | 0.071 | 0 | 0 |
| 60 | 0.052 | 0.022 | 0 | 0.095 | 0 | 0 | 0 |
| 66 | 0.023 0 | 0.009 0 | 0 | 0 | 0 | 0 | 0 |
| 70 | 0 | 0 | 0 | 0 | 0 | 0 | 0 |
| 80 | 0 | 0 | 0 | 0 | 0 | 0 | 0 |
| 90 | 0 | 0 | 0 | 0 | 0 | 0 | 0 |
| 100 | | | 0 | 0 | 0 | 0 | 0 |

**Table 2.** Concentration of $Br_2$ in aqueous electrolyte solutions depending on SoC (mixture of electrolyte) and chosen [BCA]Br in mol $L^{-1}$.

| SoC (%) | [C2Py]Br (aq) | [C4Py]Br (aq) | [C6Py]Br (aq) | [C2MIm]Br (aq) | [C3MIm]Br (aq) | [C4MIm]Br (aq) | [C6MIm]Br (aq) |
|---|---|---|---|---|---|---|---|
| 10 | 0.129 | 0.039 | 0.010 | 0.065 | 0.063 | 0.035 | 0.009 |
| 20 | 0.123 | 0.038 | 0.007 | 0.073 | 0.059 | 0.032 | 0.005 |
| 30 | 0.121 | 0.051 | 0.009 | 0.105 | 0.063 | 0.034 | 0.008 |
| 33 | 0.121 | 0.044 | 0.011 | 0.116 | 0.066 | 0.039 | 0.009 |
| 40 | 0.129 | 0.076 | 0.030 | 0.136 | 0.075 | 0.056 | 0.019 |
| 50 | 0.150 | 0.106 | 0.098 | 0.140 | 0.103 | 0.089 | 0.063 |
| 60 | 0.181 | 0.165 | 0.155 | 0.137 | 0.169 | 0.163 | 0.153 |
| 66 | 0.214 | 0.197 | 0.159 | 0.162 | 0.189 | 0.204 | 0.146 |
| 70 | 0.261 | 0.211 | 0.184 | 0.182 | 0.211 | 0.213 | 0.179 |
| 80 | 0.263 | 0.277 | 0.228 | 0.244 | 0.263 | 0.271 | 0.246 |
| 90 | 0.257 | 0.276 | 0.260 | 0.309 | 0.284 | 0.310 | 0.273 |
| 100 | 0.262 | 0.227 | 0.217 | 0.333 | 0.264 | 0.279 | 0.280 |

**Table S3.** $Br_2$ distribution in different polybromides: fraction of $Br_2$ in tribromide $Br_3^-$.

| SoC (%) | [C2Py]Br (aq) | [C4Py]Br (aq) | [C6Py]Br (aq) | [C2MIm]Br (aq) | [C3MIm]Br (aq) | [C4MIm]Br (aq) | [C6MIm]Br (aq) |
|---|---|---|---|---|---|---|---|
| 10 | 0.329 | 0.364 | 0.485 | 0.342 | 0.319 | 0.349 | 0.499 |
| 20 | 0.337 | 0.269 | 0.467 | 0.338 | 0.312 | 0.335 | 0.455 |
| 30 | 0.323 | 0.269 | 0.385 | 0.306 | 0.299 | 0.329 | 0.425 |
| 33 | 0.329 | 0.282 | 0.404 | 0.305 | 0.303 | 0.258 | 0.435 |

| | | | | | | | |
|---|---|---|---|---|---|---|---|
| 40 | 0.317 | 0.307 | 0.292 | 0.306 | 0.314 | 0.315 | 0.379 |
| 50 | 0.314 | 0.318 | 0.322 | 0.308 | 0.304 | 0.291 | 0.308 |
| 60 | 0.334 | 0.326 | 0.333 | 0.335 | 0.309 | 0.311 | 0.307 |
| 66 | 0.343 | 0.329 | 0.358 | 0.339 | 0.320 | 0.317 | 0.330 |
| 70 | 0.344 | 0.345 | 0.357 | 0.342 | 0.326 | 0.354 | 0.324 |
| 80 | 0.3654 | 0.372 | 0.372 | 0.351 | 0.349 | 0.354 | 0.352 |
| 90 | 0.408 | 0.362 | 0.427 | 0.364 | 0.383 | 0.420 | 0.376 |
| 100 | 0.445 | 0.480 | 0.473 | 0.403 | 0.425 | 0.466 | 0.416 |

**Table 4.** $Br_2$ distribution in different polybromides: fraction of $Br_2$ in pentabromide $Br_5^-$.

| SoC (%) | [C2Py]Br (aq) | [C4Py]Br (aq) | [C6Py]Br (aq) | [C2MIm]Br (aq) | [C3MIm]Br (aq) | [C4MIm]Br (aq) | [C6MIm]Br (aq) |
|---|---|---|---|---|---|---|---|
| 10 | 0.587 | 0.636 | 0.515 | 0.582 | 0.587 | 0.651 | 0.501 |
| 20 | 0.595 | 0.587 | 0.533 | 0.591 | 0.584 | 0.665 | 0.545 |
| 30 | 0.596 | 0.562 | 0.615 | 0.594 | 0.565 | 0.671 | 0.574 |
| 33 | 0.594 | 0.567 | 0.596 | 0.612 | 0.581 | 0.653 | 0.565 |
| 40 | 0.593 | 0.609 | 0.540 | 0.606 | 0.557 | 0.685 | 0.621 |
| 50 | 0.587 | 0.599 | 0.589 | 0.597 | 0.575 | 0.620 | 0.555 |
| 60 | 0.576 | 0.564 | 0.558 | 0.585 | 0.571 | 0.604 | 0.571 |
| 66 | 0.571 | 0.556 | 0.555 | 0.575 | 0.568 | 0.585 | 0.571 |
| 70 | 0.564 | 0.550 | 0.542 | 0.570 | 0.557 | 0.646 | 0.559 |
| 80 | 0.540 | 0.530 | 0.544 | 0.560 | 0.539 | 0.565 | 0.544 |
| 90 | 0.515 | 0.505 | 0.520 | 0.546 | 0.515 | 0.580 | 0.519 |
| 100 | 0.472 | 0.472 | 0.449 | 0.508 | 0.479 | 0.534 | 0.480 |

**Table 5.** $Br_2$ distribution in different polybromides: fraction of $Br_2$ in heptabromide $Br_7^-$.

| SoC (%) | [C2Py]Br (aq) | [C4Py]Br (aq) | [C6Py]Br (aq) | [C2MIm]Br (aq) | [C3MIm]Br (aq) | [C4MIm]Br (aq) | [C6MIm]Br (aq) |
|---|---|---|---|---|---|---|---|
| 10 | 0.084 | 0 | 0 | 0.076 | 0.094 | 0 | 0 |
| 20 | 0.068 | 0.144 | 0 | 0.072 | 0.104 | 0 | 0 |
| 30 | 0.081 | 0.169 | 0 | 0.099 | 0.136 | 0 | 0 |
| 33 | 0.077 | 0.151 | 0 | 0.083 | 0.115 | 0.090 | 0 |
| 40 | 0.090 | 0.084 | 0.168 | 0.088 | 0.129 | 0 | 0 |
| 50 | 0.099 | 0.082 | 0.089 | 0.095 | 0.121 | 0.089 | 0.137 |

| | | | | | | | |
|---|---|---|---|---|---|---|---|
| 60 | 0.090 | 0.110 | 0.109 | 0.080 | 0.120 | 0.084 | 0.122 |
| 66 | 0.088 | 0.115 | 0.087 | 0.086 | 0.112 | 0.098 | 0.110 |
| 70 | 0.092 | 0.106 | 0.101 | 0.088 | 0.117 | 0 | 0.116 |
| 80 | 0.095 | 0.098 | 0.083 | 0.089 | 0.112 | 0.081 | 0.104 |
| 90 | 0.077 | 0.133 | 0.053 | 0.090 | 0.101 | 0 | 0.105 |
| 100 | 0.083 | 0.048 | 0.078 | 0.089 | 0.096 | 0 | 0.104 |

**Table 6.** Electrolyte conductivity of pure $HBr/Br_2/H_2O$ electrolyte depending on SoC (mixture of electrolyte) in mS $cm^{-1}$ of ref. [8].

| SoC (%) | Conductivity (mS $cm^{-1}$) |
|---|---|
| 0 | 702.97 |
| 5 | 747.35 |
| 10 | 763.23 |
| 15 | 758.72 |
| 20 | 763.23 |
| 25 | 760.54 |
| 30 | 753.09 |
| 35 | 756.27 |
| 40 | 741.18 |
| 45 | 729.14 |
| 50 | 713.21 |
| 55 | 692.57 |
| 60 | 670.17 |
| 65 | 625.78 |
| 70 | 583.74 |
| 75 | 546.43 |
| 80 | 497.47 |
| 85 | 430.99 |
| 90 | 391.80 |
| 95 | 352.91 |

| | |
|---|---|
| 100 | 297.95 |

**Table 7.** Electrolyte conductivity of aqueous electrolyte solutions depending on state of charge (electrolyte mixture) and chosen BCA at ϑ = 23 ± 1 °C in mS cm$^{-1}$.

| SoC (%) | [C2Py]Br (aq) | [C4Py]Br (aq) | [C6Py]Br (aq) | [C2MIm]Br (aq) | [C3MIm]Br (aq) | [C4MIm]Br (aq) | [C6MIm]Br (aq) |
|---|---|---|---|---|---|---|---|
| 0 | 461.05 | 413.95 | 381.11 | 508.39 | 401.12 | 415.61 | 406.95 |
| 10 | 505.04 | 473.77 | 450.48 | 522.70 | 441.11 | 447.99 | 465.63 |
| 20 | 570.95 | 572.62 | 550.43 | 576.05 | 506.77 | 516.71 | 535.26 |
| 30 | 636.18 | 674.73 | 687.51 | 629.40 | 573.83 | 556.62 | 677.96 |
| 33 | 648.39 | 672.42 | 708.90 | 665.88 | 608.33 | 586.44 | 670.51 |
| 40 | 684.63 | 715.10 | 743.31 | 698.15 | 637.80 | 654.54 | 706.20 |
| 50 | 711.99 | 728.93 | 745.00 | 730.93 | 678.17 | 694.47 | 684.81 |
| 60 | 705.83 | 714.06 | 720.89 | 670.92 | 685.83 | 701.32 | 740.48 |
| 66 | 687.01 | 692.86 | 699.79 | 713.15 | 662.95 | 688.60 | 706.20 |
| 70 | 668.75 | 674.27 | 678.47 | 701.05 | 656.18 | 672.73 | 711.55 |
| 80 | 595.95 | 603.29 | 605.15 | 615.14 | 595.41 | 617.96 | 619.47 |
| 90 | 486.49 | 494.09 | 483.13 | 530.29 | 503.51 | 517.41 | 562.90 |
| 100 | 335.79 | 324.78 | 335.79 | 402.16 | 368.40 | 382.00 | 422.29 |

**Table 8.** Redox potential of the aqueous electrolyte phase on glassy carbon vs. NHE at ϑ = 23 ± 1 °C in V.

| **SoC (%)** | **[C2Py]Br (aq)** | **[C4Py]Br (aq)** | **[C6Py]Br (aq)** | **[C2MIm]Br (aq)** | **[C3MIm]Br (aq)** | **[C4MIm]Br (aq)** | **[C6MIm]Br (aq)** |
|---|---|---|---|---|---|---|---|
| 0 | 0.849 | 0.659 | 0.759 | -- | 0.886 | 0.850 | 0.775 |
| 10 | 0.836 | 0.824 | 0.806 | 0.827 | 0.826 | 0.820 | 0.805 |
| 20 | 0.850 | 0.836 | 0.819 | 0.844 | 0.837 | 0.833 | 0.819 |
| 30 | 0.867 | 0.857 | 0.838 | 0.863 | 0.856 | 0.852 | 0.839 |
| 33 | 0.870 | 0.858 | 0.842 | 0.866 | 0.857 | 0.857 | 0.844 |
| 40 | 0.880 | 0.875 | 0.864 | 0.876 | 0.871 | 0.872 | 0.860 |
| 50 | 0.901 | 0.898 | 0.894 | 0.890 | 0.891 | 0.894 | 0.891 |
| 60 | 0.923 | 0.928 | 0.922 | 0.903 | 0.916 | 0.920 | 0.919 |
| 66 | 0.938 | 0.936 | 0.933 | 0.912 | 0.928 | 0.934 | 0.931 |
| 70 | 0.946 | 0.944 | 0.940 | 0.920 | 0.934 | 0.944 | 0.940 |
| 80 | 0.970 | 0.920 | 0.965 | 0.937 | 0.958 | 0.968 | 0.964 |
| 90 | 0.998 | 0.999 | 0.995 | 0.962 | 0.985 | 1.000 | 0.994 |
| 100 | 1.039 | 1.059 | 1.033 | 0.994 | 1.019 | 1.049 | 1.045 |

## 5. Raman Spectra of Aqueous Electrolyte Phases for Different BCAs and the Entire SoC Range

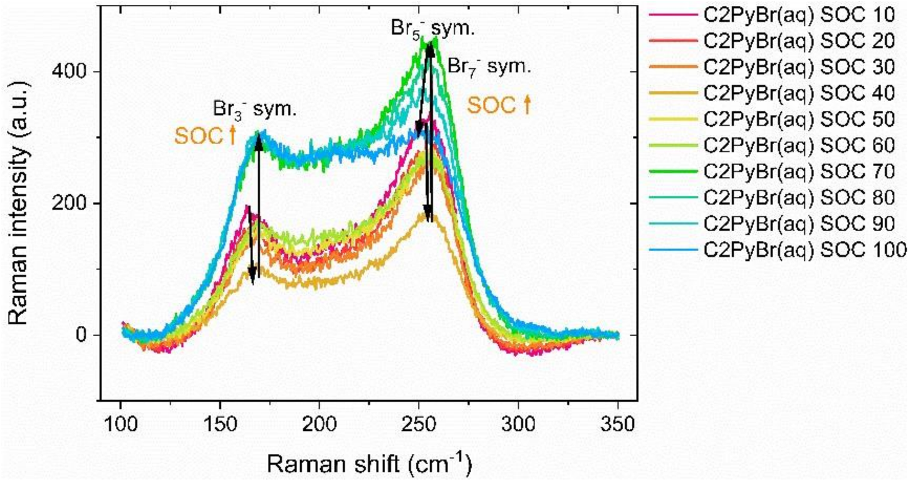


**Figure 3.** Raman spectra of aqueous electrolyte phases with [C2Py]Br as BCA as a function of state of charge (SoC). Due to low intensities, the presence of bromine $Br_2$ in aqueous solution can generally be observed. For 10 % ≤ SoC ≤ 40 %, the area below the spectra generally decreases and then increases again for an SoC of ≥ 40 %. For an SoC of ≥ 70 %, the peak at $\tilde{\nu}$ = 260 $cm^{-1}$ decreases due to low solubility of higher polybromides $Br_5^-$ and $Br_7^-$ in the aqueous electrolyte phase [8]. In comparison with the $Br_2$ concentrations in the manuscript (Figure 6), this trend shows an agreement in the concentration of $Br_2$ in the aqueous solution over the SoC range.

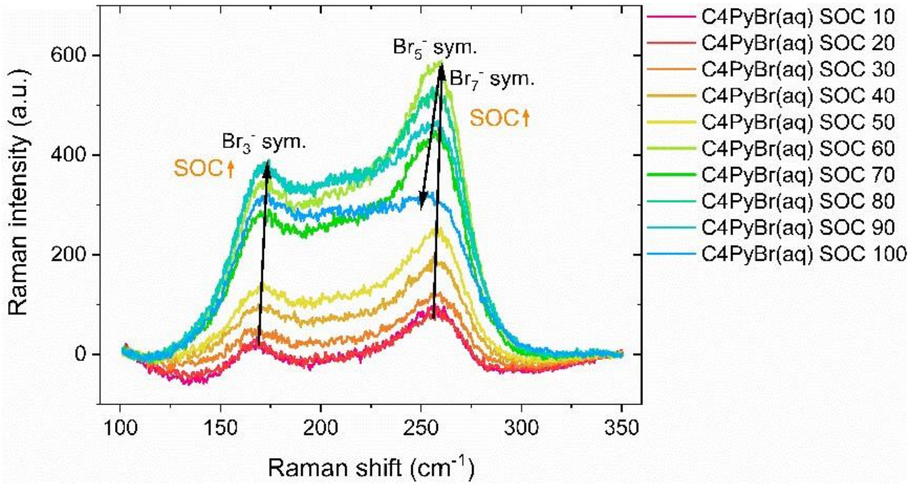


**Figure 4.** Raman spectra of aqueous electrolyte phases with [C4Py]Br as BCA as a function of state of charge (SoC). Due to low intensities, the presence of bromine $Br_2$ in the aqueous solution can generally be observed. For 10 % ≤ SoC ≤ 60 %, the area below the spectra lines generally increases, which shows that the amount of dissolved $Br_2$ in solution increases. For an SoC of > 60 %, the peak at $\tilde{\nu}$ = 260 $cm^{-1}$ decreases due to the low solubility of the higher polybromides $Br_5^-$ and $Br_7^-$ in the aqueous electrolyte phase [8]. In comparison with $Br_2$ concentrations in the manuscript (Figure 6), this trend shows an agreement in the concentration of $Br_2$ in the aqueous solution over the SoC range.

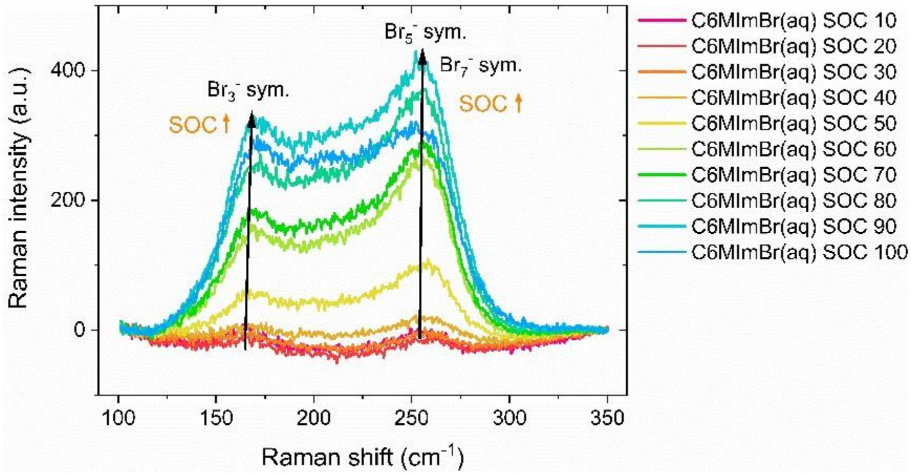


**Figure 5.** Raman spectra of aqueous electrolyte phases with [C6Py]Br as BCA as a function of state of charge (SoC). Due to low intensities the presence of bromine $Br_2$ in the aqueous solution can generally be observed. For 10 % ≤ SoC ≤ 90 %, the area below the spectra generally increases, which shows an increasing amount of dissolved $Br_2$ in solution. For an SoC of 100 %, the peak at $\tilde{v}$ = 260 cm-1 decreases due to low solubility of higher polybromides $Br_5^-$ and $Br_7^-$ in aqueous electrolyte phase [8]. In comparison with $Br_2$ concentrations in the manuscript (Figure 6), this trend shows that concentration of $Br_2$ in the aqueous solution is consistent throughout the SoC range.

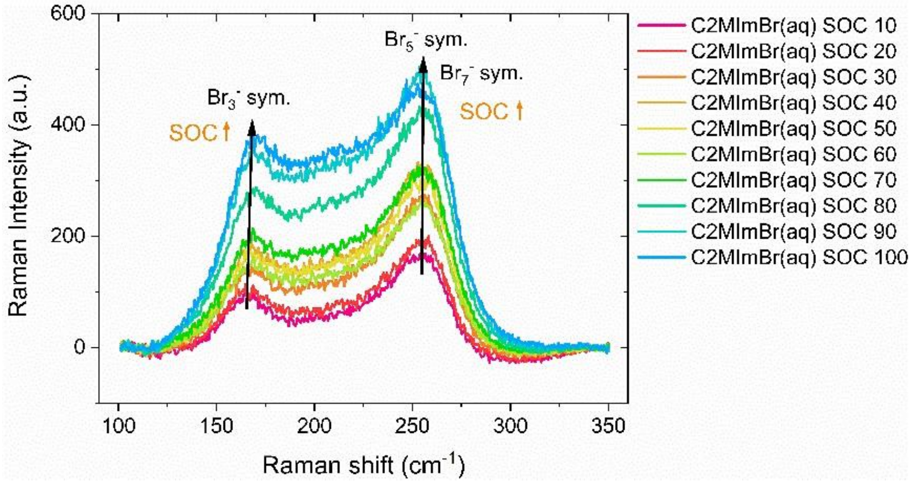


**Figure 6.** Raman spectra of aqueous electrolyte phases with [C2MIm]Br as BCA depending on state of charge (SoC). Due to low intensities, the presence of bromine $Br_2$ in the aqueous solution can generally be observed. For 10 % ≤ SoC ≤ 90 %, the area below the spectra generally increases, which shows an increasing amount of dissolved $Br_2$ in solution. In comparison with the $Br_2$ concentrations in the manuscript (Figure 6), this trend shows a correspondence in concentration of $Br_2$ in the aqueous solution over the SoC range.

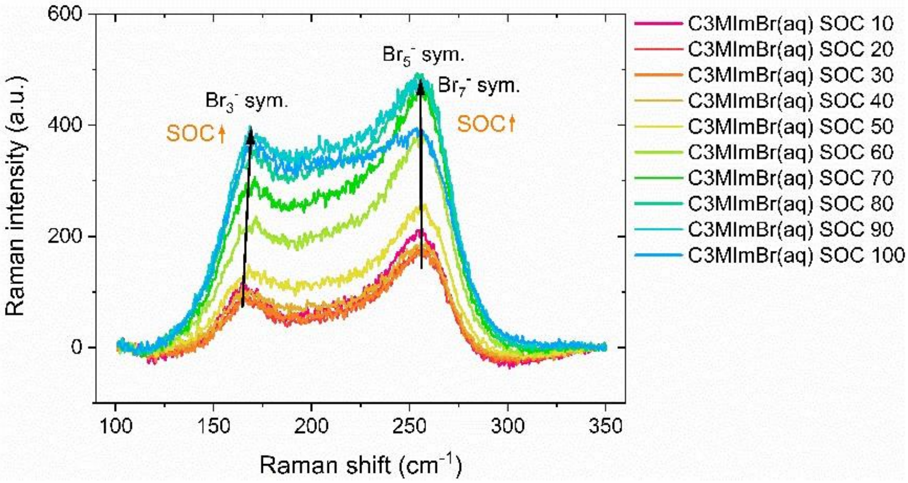


**Figure 7.** Raman spectra of aqueous electrolyte phases with [C3MIm]Br as BCA depending on state of charge (SoC). Due to low intensities, the presence of bromine $Br_2$ in the aqueous solution can generally be observed. For 10 % ≤ SoC ≤ 90 %, the area below the spectra generally increases, which shows an increasing amount of dissolved $Br_2$ in solution. For an SoC of 100 %, the peak decreases at $\tilde{v}$ = 260 $cm^{-1}$, due to low solubility of higher polybromides $Br_5^-$ and $Br_7^-$ in aqueous electrolyte phase [8]. In comparison with the $Br_2$ concentrations in the manuscript (Figure 6), this trend shows a correspondence in concentration of $Br_2$ in the aqueous solution over the SoC range.

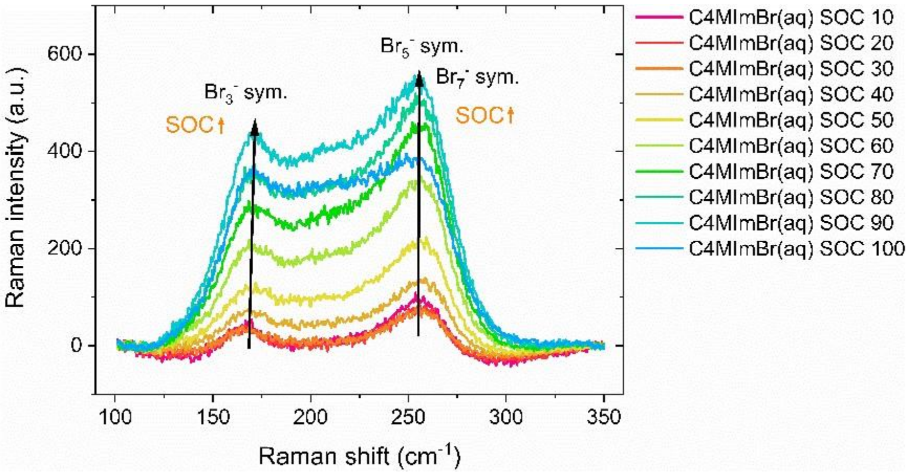


**Figure 8.** Raman spectra of aqueous electrolyte phases with [C4MIm]Br as BCA depending on state of charge (SoC). Due to low intensities, the presence of bromine $Br_2$ in the aqueous solution can generally be observed. For 10 % SoC ≤ 90 %, the area below the spectra generally increases, which shows an increasing amount of dissolved $Br_2$ in solution. For an SoC of 100 %, the peak decreases at $\tilde{v}$ = 260 $cm^{-1}$ due to the low solubility of the higher polybromides $Br_5^-$ and $Br_7^-$ in the aqueous electrolyte phase [8]. In comparison with the $Br_2$ concentrations in the manuscript (Figure 6), this trend shows a correspondence in concentration of $Br_2$ in the aqueous solution over the SoC range.

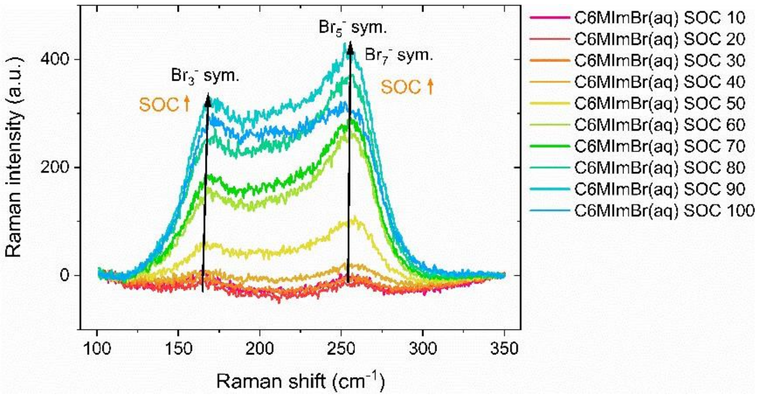


**Figure 9.** Raman spectra of aqueous electrolyte phases with [C6MIm]Br as BCA depending on state of charge (SoC). Due to low intensities, the presence of bromine $Br_2$ in the aqueous solution can generally be observed. For 10 % ≤ SoC ≤ 90 %, the area below the spectra generally increases, which shows an increasing amount of dissolved $Br_2$ in solution. For an SoC of 100 %, the peak decreases at $\tilde{\nu}$ = 260 $cm^{-1}$ due to low solubility of higher polybromides $Br_5^-$ and $Br_7^-$ in the aqueous electrolyte phase [8]. In comparison with $Br_2$ concentrations in the manuscript (Figure 6), this trend shows that concentration of $Br_2$ in the aqueous solution is consistent over the SoC range.

## References for SM


1. Sashina, E.S.; Kashirskii, D.A.; Janowska, G.; Zaborski, M. Thermal properties of 1-alkyl-3-methylpyridinium halide-based ionic liquids. *Thermochim. Acta* **2013**, *568*, 185–188, doi:10.1016/j.tca.2013.06.022.
2. Aupoix, A.; Pégot, B.; Vo-Thanh, G. Synthesis of imidazolium and pyridinium-based ionic liquids and application of 1-alkyl-3-methylimidazolium salts as pre-catalysts for the benzoin condensation using solvent-free and microwave activation. *Tetrahedron* **2010**, *66*, 1352–1356, doi:10.1016/j.tet.2009.11.110.
3. Dzyuba, S.V.; Bartsch, R.A. Efficient synthesis of 1-alkyl(aralkyl)-3-methyl(ethyl)imidazolium halides: Precursors for room-temperature ionic liquids. *J. Heterocycl. Chem.* **2001**, *38*, 265–268, doi:10.1002/jhet.5570380139.
4. Bonhôte, P.; Dias, A.-P.; Papageorgiou, N.; Kalyanasundaram, K.; Grätzel, M. Hydrophobic, Highly Conductive Ambient-Temperature Molten Salts †. *Inorg. Chem.* **1996**, *35*, 1168–1178, doi:10.1021/ic951325x.
5. Alexey Deyko; Stephen G Hessey; Peter Licence; Elena A Chernikova; Robert G Jones. The enthalpies of vaporisation of ionic liquids: New measurements and predictions. *PCCP* **2012**, *14*, 3181–3193, doi:10.1039/c2cp23705a.
6. Butkene, R.V.; Mikul'skene, G.V.; ikher-Lorka, O.S.; Kupyatis, G.-K.K. Alkylation of 4-picolinium salts under phase transfer conditions. *Chem. Heterocycl. Compd.* **1989**, *25*, 433–438, doi:10.1007/bf00480760.
7. Ghosh, R.; Ekka, D.; Rajbanshi, B.; Yasmin, A.; Roy, M.N. Synthesis,characterization of 1-butyl-4methylpyridinium lauryl sulfate and its inclusion phenomenon with β-cyclodextrin for enhanced applications. *Colloids Surf., A* **2018**, *548*, 206–217, doi:10.1016/j.colsurfa.2018.01.003.
8. Küttinger, M.; Wlodarczyk, J.K.; Daubner, D.; Fischer, P.; Tübke, J. High energy density electrolytes for H 2 /Br 2 redox flow batteries, their polybromide composition and influence on battery cycling limits. *RSC Adv.* **2021**, *11*, 5218–5229, doi:10.1039/D0RA10721B.
9. Easton, M.E.; Ward, A.J.; Hudson, T.; Turner, P.; Masters, A.F.; Maschmeyer, T. The formation of high-order polybromides in a room-temperature ionic liquid: from monoanions ([Br5 ](-) to [Br11 ](-) ) to the isolation of [PC16 H36 ]2 [Br24 ] as determined by van der Waals Bonding Radii. *Chem. Eur. J.* **2015**, *21*, 2961–2965, doi:10.1002/chem.201404505.
10. Chen, X.; Rickard, M.A.; Hull, J.W.; Zheng, C.; Leugers, A.; Simoncic, P. Raman spectroscopic investigation of tetraethylammonium polybromides. *Inorg. Chem.* **2010**, *49*, 8684–8689, doi:10.1021/ic100869r.
11. Kanno, H. Raman study of aqueous HX solutions (X = F, Cl, Br and I) in both liquid and glassy states. *J. Raman Spectrosc.* **1993**, *24*, 689–693, doi:10.1002/jrs.1250241009.
12. Kanno, H.; Hiraishi, J. Existence of H3O+ ions in glassy aqueous HX solutions (X = Cl and Br). *Chem. Phys. Lett.* **1984**, *107*, 438–441, doi:10.1016/S0009-2614(84)80251-1.
13. Walrafen, G.E. Raman Spectral Studies of Water Structure. *The Journal of Chemical Physics* **1964**, *40*, 3249– 3256, doi:10.1063/1.1724992.
14. Ligny, D. de; Guillaud, E.; Gailhanou, H.; Blanc, P. Raman Spectroscopy of Adsorbed Water in Clays: First Attempt at Band Assignment. *Procedia Earth Planet. Sci.* **2013**, *7*, 203–206, doi:10.1016/j.proeps.2013.03.202.
15. Schrader, B.; Meier, W. *Raman/IR Atlas organischer Verbindungen/of Organic Compounds;* Verlag Chemie: Weinheim/Bergstr., 1974.
16. Fleischmann, M.; Hendra, P.J.; McQuillan, A.J. Raman spectra of pyridine adsorbed at a silver electrode. *Chem. Phys. Lett.* **1974**, *26*, 163–166, doi:10.1016/0009-2614(74)85388-1.
17. Cook, D. Vibrational spectra of pyridinium salts. *Can. J. Chem.* **1961**, *39*, 2009–2024, doi:10.1139/v61-271.

18. Treimer, S.; Tang, A.; Johnson, D.C. A Consideration of the Application of Koutecký-Levich Plots in the Diagnoses of Charge-Transfer Mechanisms at Rotated Disk Electrodes. *Electroanalysis* **2002**, *14*, 165, doi:10.1002/1521-4109(200202)14:3<165:AID-ELAN165>3.0.CO;2-6.
19. Bard, A.J.; Faulkner, L.R. *Electrochemical Methods*: *Fundamentals and Applications*. Second Edition, 2nd edition; John Wiley & Sons, Inc.: New York, NY, United States of America, 2001, ISBN 978-0-471-04372.
20. Vielstich, W. Theorie und Anwendung der rotierenden Scheibenelektrode. *Z. Anal. Chem.* **1960**, *173*, 84–87, doi:10.1007/BF00448719.
21. Frumkin, A.; Nekrasov, L.; Levich, B.; Ivanov, J. Die Anwendung der rotierenden Scheibenelektrode mit einem Ringe zur Untersuchung von Zwischenprodukten elektrochemischer Reaktionen. *Journal of Electroanalytical Chemistry (1959)* **1959**, *1*, 84–90, doi:10.1016/0022-0728(59)80012-7.
22. Easton, M.E.; Ward, A.J.; Chan, B.; Radom, L.; Masters, A.F.; Maschmeyer, T. Factors influencing the formation of polybromide monoanions in solutions of ionic liquid bromide salts. *Phys. Chem. Chem. Phys.* **2016**, *18*, 7251–7260, doi:10.1039/c5cp06913k.
23. Haller, H.; Schröder, J.; Riedel, S. Structural evidence for undecabromide [Br11]-. *Angew. Chem. Int. Ed.* **2013**, *52*, 4937–4940, doi:10.1002/anie.201209928.
24. Hayward, G.C.; Hendra, P.J. The far infra-red and Raman spectra of the trihalide ions IBr−2 and I−3.
25. *Spectrochimica Acta Part A: Molecular Spectroscopy* **1967**, *23*, 2309–2314, doi:10.1016/0584-8539(67)80124-7.
26. Evans, J.C.; Lo, Grace Y. S. Vibrational spectra of BrO-, BrO2-, Br3-, and Br5-. *Inorg. Chem.* **1967**, *6*, 1483–1486, doi:10.1021/ic50054a011.
27. Bauer, G.; Drobits, J.; Fabjan, C.; Mikosch, H.; Schuster, P. Raman spectroscopic study of the bromine storing complex phase in a zinc-flow battery. *J. Electroanal. Chem.* **1997**, *427*, 123–128, doi:10.1016/S00220728(96)04992-3.
28. *CRC handbook of chemistry and physics*: *A ready-reference book of chemical and physical data;* Haynes, W.M.; Lide, D.R., Eds., 96. ed., 2015-2016; CRC Press: Boca Raton, Fla., 2015, ISBN 978-1482260960.
29. Shimizu, Y.; Wachi, Y.; Fujii, K.; Imanari, M.; Nishikawa, K. NMR Study on Ion Dynamics and Phase Behavior of a Piperidinium-Based Room-Temperature Ionic Liquid: 1-Butyl-1-methylpiperidinium Bis(fluorosulfonyl)amide. *J. Phys. Chem. B* **2016**, *120*, 5710–5719, doi:10.1021/acs.jpcb.6b04095.
30. Yim, T.; Lee, H.Y.; Kim, H.-J.; Mun, J.; Kim, S.; Oh, S.M.; Kim, Y.G. Synthesis and Properties of Pyrrolidinium and Piperidinium Bis(trifluoromethanesulfonyl)imide Ionic Liquids with Allyl Substituents. *Bull. Korean Chem. Soc.* **2007**, *28*, 1567–1572, doi:10.5012/bkcs.2007.28.9.1567.
